# Incubation determines favorable microbial communities in Chinese alligator nests

**DOI:** 10.3389/fmicb.2022.983808

**Published:** 2022-10-13

**Authors:** Qin-Zhang Yu, Meng-Yuan Hu, Li Wang, Jian-Qing Lin, Sheng-Guo Fang

**Affiliations:** ^1^The Key Laboratory of Conservation Biology for Endangered Wildlife of the Ministry of Education, State Conservation Center for Gene Resources of Endangered Wildlife, College of Life Sciences, Zhejiang University, Hangzhou, China; ^2^Guangdong Provincial Key Laboratory of Marine Disaster Prediction and Prevention, Guangdong Provincial Key Laboratory of Marine Biotechnology, Institute of Marine Science, Shantou University, Shantou, China

**Keywords:** nest material, bacteria, fungi, incubation, dynamics, Chinese alligator

## Abstract

Nest materials are a major heat source due to rotting promoted by microbial activity. Additionally, they are a potential microbial source given their direct contact with eggshells. Microbial dynamics during incubation have been studied in wild birds; however, similar studies in reptiles remain elusive. Here, the study characterized microbial communities in the nest materials of Chinese alligator (*Alligator sinensis*) using high-throughput sequencing of bacterial 16S rRNA genes and fungal internal transcribed spacer (ITS) region sequences. The results showed that significant changes in the diversity and structure of microbial communities according to different incubation periods. The diversity and richness of bacterial species increased significantly over time, but the relative abundance of the most dominant bacteria in pre-incubation period, including some pathogenic bacteria, declined after incubation. In contrast, fungal species diversity and richness decreased significantly with time. Additionally, nest material composition significantly influenced microbial community structure rather than species diversity and richness. Notably, the fungal community structure showed a stronger response than bacteria to nest material composition, which varied due to differences in plant litter composition. Our results demonstrate the significant response of microbial community diversity and structure to differences in incubation periods and nest material composition in reptiles. It is further emphasized that the importance of incubation period in the conservation of the Chinese alligator and could inform similar studies in other reptiles and birds.

## Introduction

Reproduction is one of the most critical stages of the animal life cycle and involves the transmission of individual genes for the continuation of the population. Reproductive activity requires a stable and safe environment to guarantee successful breeding, and nests play a critical role in determining the breeding success of especially egg-laying species (Hanmer et al., 2017). Reptiles typically deposit their eggs in an untended nest (Noble et al., 2018), and nest-site selection can directly influence hatching success, survival, phenotype, and sex ratio (Hoi et al., 1994, 1996; Weidinger, 2002; Mitchell et al., 2013). All crocodilians undergo temperature-dependent sex determination (TSD), in which the sex of embryos is determined during incubation by the action of temperature on the sexual differentiation system during a thermo-sensitive period (TSP) (Lang and Andrews, 1994). The nests of many crocodile species have been shown to maintain warm and relatively stable temperatures despite ambient fluctuations (Magnusson, 1979). The major heat sources in nests include environmental temperatures, embryo metabolism, termite mounds, rotting vegetation, and microbial metabolism (Coenen-Strass et al., 1980; Magnusson et al., 1985). In particular, significant amounts of heat are produced in the nest by rotting vegetation (Tansey, 1973; Magnusson, 1979). In wood ants, the heat production of nest material originates from microbial activity and is chiefly the result of aerobic metabolism (Coenen-Strass et al., 1980). However, how nest material and microbes influence nest temperature in crocodiles and alligators remains unclear.

Birds and reptiles often use a wide variety of plant litter, soil, animal and artificial materials to construct their nests; thus, differences in nest material composition can influence microbial diversity and structure, in turn influencing overall metabolic activity. Birds prefer to use aromatic plants and feathers for nest building because of the antimicrobial properties of some volatile compounds produced by green plants and chemicals of feather-degrading bacteria (Ruiz-Castellano et al., 2016, 2019). European Starlings (*Sturnus vulgaris*) use fresh vegetation that release anti-pathogenic compounds as a nesting material to inhibit bacterial growth (Clark and Mason, 1985), and fewer bacteria were sampled in nests with herbs than those without (Gwinner and Berger, 2005). House finches (*Haemorhous mexicanus*) use cigarette butts as part of the nesting material as the cigarette reduces parasite load and increases breeding success (Suárez-Rodríguez et al., 2013). Chinese alligators (*Alligator sinensis*) select nesting sites based on environmental factors (López-Luna et al., 2015), though whether a similar preference in the selection of nest material exists for these species is unclear.

Bacteria and fungi quickly develop on and colonize nest material. Eggshells are in direct contact with nest material during incubation and, as a result, are sensitive to the nest’s microbiome. Many studies on birds and turtles have shown a positive association between eggshell and nest material microbial communities (Martínez-García et al., 2016; Ruiz-Castellano et al., 2016; Ackerman, 2017; Candan and Candan, 2020). Some bacteria and fungi can digest the cuticle layer and penetrate the eggshell through pores, ultimately reducing clutch success (Kozlowski et al., 1991; Bruce and Drysdale, 1994; Houston et al., 1997; Cook et al., 2003, 2005a,2005b). Microbial communities fluctuate during incubation. A study on the Oriental Tit suggested that bacterial communities had higher diversity and some pathogenic bacteria had a lower relative abundance (Song et al., 2022). In addition, a decrease in the number of potentially harmful gram-negative bacteria, and complete extinction of harmful hemolytic bacteria can be observed after incubation, the total microbial abundance, diversity, and growth decreases after incubation (Cook et al., 2005a,b; Potter et al., 2013; Brandl et al., 2014; Grizard et al., 2014, 2015; Lee et al., 2014). Nest materials also mediate microbial transmission between parents and offspring. For example, the early life assembly of passerine chick gut microbiota is shaped by maternal gut microbes via the nest (Brandl et al., 2014; Chen et al., 2020). Thus, the composition and dynamics of microbial communities in nest materials could further elucidate studies of breeding success and thus be vital to conservation biology. Here, we examined the microbial community dynamics of different nest materials during various incubation periods in nests of the Chinese alligator using a high throughput sequencing technique. Our primary aims are to test (1) Whether there are significant dynamics in bacterial and fungal diversity and structure during different incubation and different nest material composition, and explore possible mechanisms based on our results; (2) discuss the potential role of nest material microbial community dynamics in reptile growth and adaption.

## Materials and methods

### Field site and nest material sampling

**FIGURE 1:**
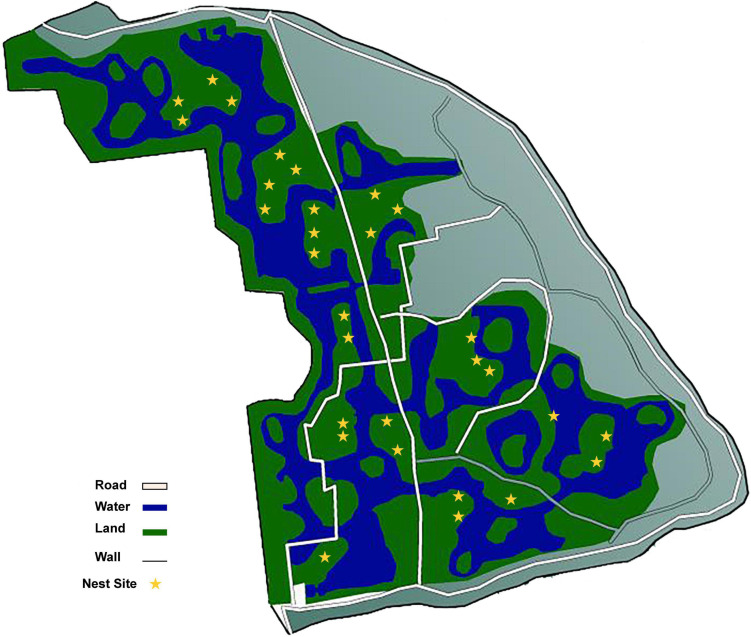
The sampling sites in this research.

The study site was located in the Yinjiabian Changxing Chinese Alligator Nature Reserve (YCCNR, 30°93′ N, 119°73′ E) in Zhejiang Province, China. YCCNR covers an area of 5401.57 m^2^ and contains a natural wetland (Zhao et al., 2013). During late June (pre-incubation period), late July (mid-incubation period), and late August (post-incubation period) of the nesting season in 2021, a total of 90 samples were collected from 30 nest mounds at the approximate depth of the clutch and placed in a disposable sample bag (Figure 1). A total of 12 plant litter and surface soil samples were collected randomly from the area surrounding the nests and were used as control groups. All samples were transported on ice. Nest material composition was determined based on the surrounding plants. Almost all nests consisted of mud and plant litter, including fallen leaves, branches, and weeds. Nests were divided into 3 groups according to nest material composition: (i) B groups, mainly included bamboo leaves and a little soil (B1–B3); (ii) C groups, had couch grass and a few other herbaceous plants with some soil (C1–C3); (iii) M groups, mixed plant litter and soil, including leaves and branches of some woody plants, such as camphor and osmanthus (M1-M3). Arabic numerals (1, 2, and 3) represent the pre-, mid-, and post-incubation period, respectively (Supplementary Table 1).

### DNA extraction, 16S rDNA and ITS1 gene amplification and sequencing analysis

Total genomic DNA was extracted using the *CTAB*/*SDS* method; purity and DNA concentration were determined using agarose gel electrophoresis. An appropriate amount of DNA was placed in a sterile centrifuge tube and diluted with sterile water to 1 ng/μL. The diluted genomic DNA was used as a template according to the selection of the sequencing region. Specific primers with barcodes, Phusion^®^ High-Fidelity PCR Master Mix with GC Buffer (New England Biolabs), and high-fidelity enzymes were used for polymerase chain reaction (PCR). For bacteria, the V4–V5 region of 16S rDNA was amplified using the forward primer 515F (5′-GTGCCAGCMGCCGCGGTAA-3′) and reverse primer 806R (5′-GGACTACHVGGGTWTCTAAT-3′) (Bergmann et al., 2011). For fungi, the internal transcribed spacer 1 (ITS1) was amplified using the forward primer for ITS5-1737F (5′-GGAAGTAAAAGTCGTAACAAGG-3′) and reverse primer for ITS2-2043R (5′-GCTGCGTTCTTCATCGATGC-3′) (Bellemain et al., 2010). The PCR products were subjected to electrophoresis on 2% agarose gel. A TruSeq^®^ DNA PCR-Free Sample Preparation Kit was used to construct a library, which was quantified using Qubit and Q-PCR. Sequencing was performed using a NovaSeq 6000.

### Bioinformatics and statistical analysis

According to the barcode and PCR amplification primer sequence, the sample data were separated from the down-machine data, and the barcode and primer sequences were trimmed. FLASH (v1.2.7^1^) (Magoč and Salzberg, 2011) was used to splice the reads of each sample. The raw tags obtained were stitched together under strict filtering (Bokulich et al., 2013) to obtain clean tags. To obtain effective tags, chimeric sequences were removed from the clean tags (Haas et al., 2011; Rognes et al., 2016) after QIIME quality-controlled processing (v1.9.1^2^) (Caporaso et al., 2010). Nucleotide sequences showing 97% identity were clustered into operational taxonomic units (OTUs) using Uparse (Uparse v7.0.1001^3^) (Edgar, 2013). Species annotation of the representative OTU sequence was carried out using the Mothur method and SSUrRNA database (defined threshold of 0.8–1.0) (Quast et al., 2013).

Alpha diversity indices (Observed-OTUs, Chao1, Shannon, Simpson, ACE, Goods-coverage, and PD_whole_tree), beta diversity (unweighted and weighted UniFrac distance) indices, and UPGMA sample clustering tree was calculated and constructed using QIIME (v1.9.1). The alpha and beta diversity indices were compared among samples with the Wilcoxon rank-sum test suing R software. Analysis of similarity (ANOSIM) was performed based on the Bray–Curtis distance matrix using the R vegan package. We also compared the relative abundances of bacteria and fungi at various taxonomic levels based on the linear discriminant analysis (LDA) effect size (LEfSe) method using LEfSe software (Segata et al., 2011); statistically significant differences in the relative abundance of microbiota between groups were compared with a *t*-test. Additionally, we used Tax4Fun to predict the bacterial functional pathway from 16S rRNA data. The FunGuild database was used to predict fungal function. Heat maps, box plots, and bar charts were generated using the “ggplot2” package of R software. Figure modifications were performed using Adobe Illustrator.

## Results

### Summary of 16 S rRNA gene and ITS sequencing data

A total of 8311846 high quality 16S rRNA gene reads were obtained from 90 nest material bacterial samples and 12 control groups, the average lengths 373 bp (Supplementary Table 2). The OTUs identified in all samples were divided into 81 phyla, 165 classes, 370 orders, 547 families and 1004 genera. In total, 8399134 clean reads of fungi were obtained from 89 nest materials fungal samples (eliminate abnormal sample C14) and 12 control groups, the average lengths 233 bp (Supplementary Table 3). The OTUs identified in all samples were divided into 15 phyla, 61 classes, 188 orders, 440 families and 1028 genera.3.2 Differences in microbial composition between groups.

### Differences in microbial composition between groups

**FIGURE 2:**
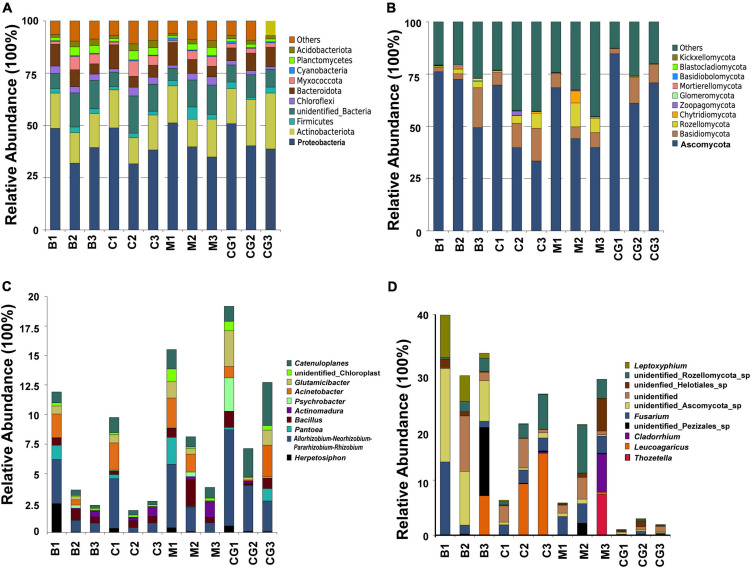
Relative abundance of microbes at the phyla and genus levels according to nest material composition and incubation period. **(A)** The dominant bacteria phyla, **(B)** dominant fungi phyla, **(C)** dominant bacterial genera, and **(D)** dominant fungi genera. The letters in group ID represents nest material composition (B, bamboo leaf; C, couch grass; M, mixed litter; CG, control group); Arabic numerals represent different incubation periods (1, pre-incubation period; 2, mid-incubation period; 3, post-incubation period).

**FIGURE 3:**
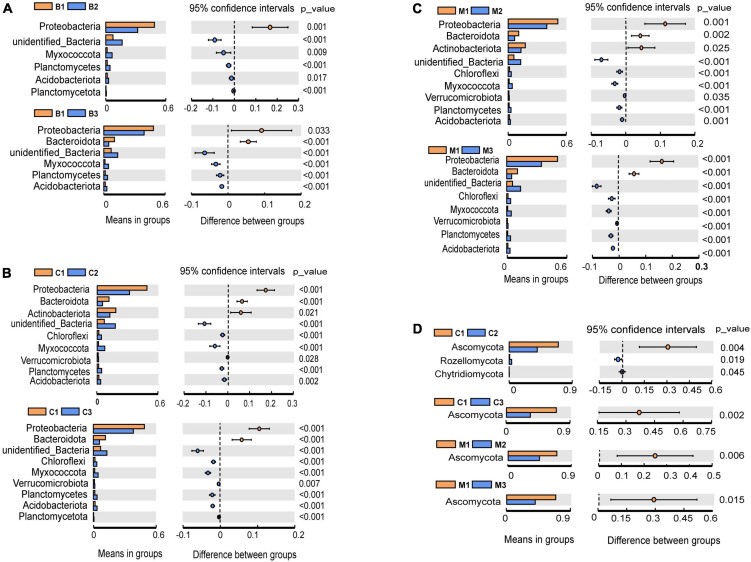
Differential abundance analysis and comparisons with *t*-test at the phylum level during incubation. **(A–C)** Bacteria; **(D)** fungi. Only significant results (*p* < 0.05) are shown. The letters in group ID represents nest material composition (B, bamboo leaf; C, couch grass; M, mixed litter; CG, control group); Arabic numerals represent different incubation periods (1, pre-incubation period; 2, mid-incubation period; 3, post-incubation period).

The most abundant bacterial phyla, Proteobacteria, Actinobacteria, Bacteroidetes, Chloroflexi, Firmicutes, Myxococcota, Planctomycetes, and Verrucomicrobia, were common to all nest material groups (B, C, and M) as well as the control group (CG) (Figure 2A). In the same incubation period, the relative abundance of the major bacterial phyla was similar between groups (B1 vs. C1 vs. M1; B2 vs. C2 vs. M2; B3 vs. C3 vs. M3) (Figure 2A). Differential abundance analysis showed that Proteobacteria and Bacteroidetes in the same nest material decreased in relative abundance after incubation, whereas Planctomycetes and Myxococcota became significantly more abundant (Figures 3A–C). The most abundant fungal phyla were Ascomycota, Basidiomycota, and Rozellomycota (Figure 2B). The relative abundance of the Ascomycota phylum was over 70% in pre-incubation period, whereas it became significantly less abundant after incubation (Figure 3D).

**FIGURE 4:**
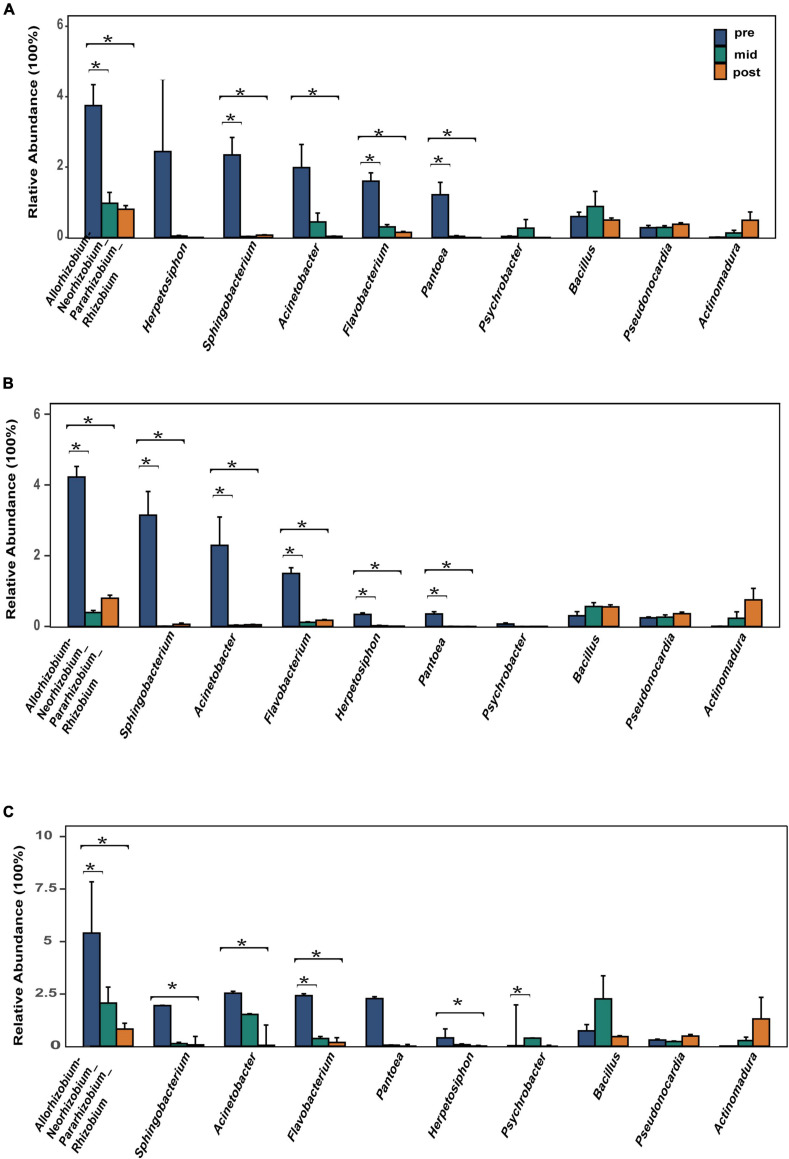
Relative abundance of the 10 most abundant bacterial genera. **(A)** Bamboo leaf groups. **(B)** Couch grass groups. **(C)** Mixed litter groups. Significant results (*p* < 0.05) from the differential abundance analysis between different incubation periods are shown as asterisks (*). Pre, pre-incubation period; mid, mid-incubation period; post, post-incubation period.

The top 10 most abundant bacterial genera were *Herpetosiphon*, *Flavobacterium*, *Allorhizobium-Neorhizobium-Pararhizobium-Rhizobium*, *Pantoea*, *Acinetobacter*, *Sphingobacterium*, *Pseudonocardia*, *Psychrobacter*, *Bacillus*, and *Actinomadura*. These bacteria genera include many pathogenic bacteria species and predatory bacteria species (Figure 2C). Differential abundance analysis of the same nest material showed a significant decrease in the abundance of the most dominant bacterial genera after incubation (Figure 4). The dominant fungal genera included *Fusarium*, *Thozetella*, *Leucoagaricus*, *Sarocladium*, *Cladorrhinum*, *Plectosphaerella*, and some unknown species. The relative abundance of the respective dominant fungal genera varied with nest material composition (Figure 2D).

Significant difference biomarkers were revealed at different levels by LEfSe analysis. The results revealed that if nest material composition was same, most of significant bacteria and fungi (C groups and M groups) all enriched at pre-incubation, difference biomarkers became less significantly after incubation (Supplementary Figures 1, 2). There were fewer biomarkers between different nest materials composition groups comparing to incubation periods changes. Additionally, most significant difference bacteria were mainly enriched at mid-incubation, while most significant difference fungal taxa were enriched during pre-incubation (Supplementary Figure 3).

### Changes in microbial diversity and structure

#### Alpha diversity analysis

**FIGURE 5:**
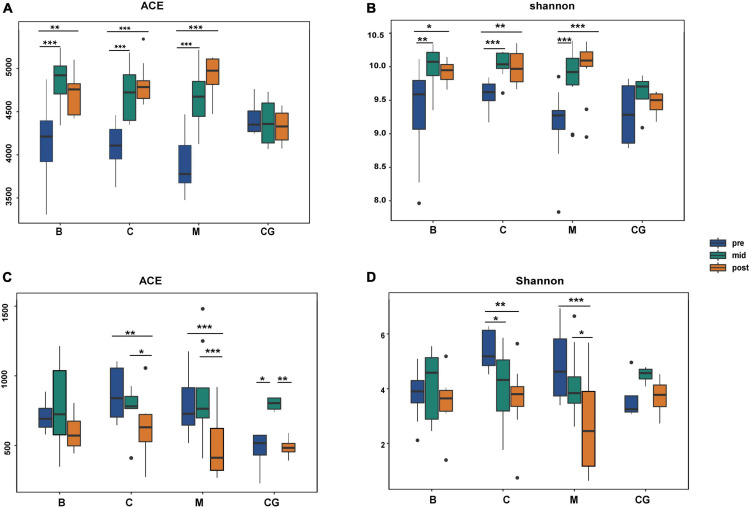
Alpha diversity of bacterial and fungal communities according to nest material composition and incubation period. **(A,B)** Bacterial alpha diversity indexes; **(C,D)** fungal alpha diversity indexes; ns: *p* > 0.05; *p < 0.05; **p < 0.01; ***p < 0.001. B, bamboo leaf; C, couch grass; M, mixed litter; pre, pre-incubation period; mid, mid-incubation period; post, post-incubation period.

Alpha diversity indices for all bacteria were higher than those for fungi, indicating a comparatively greater richness and diversity of bacteria (Supplementary Table 4). For the same nest material, bacterial and fungal alpha diversity indices varied significantly according to incubation period (*p* < 0.05; B1 vs. B2 vs. B3; C1 vs. C2 vs. C3; M1 vs. M2 vs. M3); bacterial and fungal indices showed an increasing and decreasing trend, respectively. For the same incubation period, bacterial and fungal alpha diversity indices showed no significant difference between nest material composition groups (*p* > 0.05; B1 vs. C1 vs. M1; B2 vs. C2 vs. M2; B3 vs. C3 vs. M3). In the control group, bacterial indices were stable throughout, and fungal indices peaked at mid-incubation (Figure 5 and Supplementary Table 5).

Therefore, the species diversity and richness of microbial communities showed a more robust response to changes in the incubation period than nest material composition. Bacterial communities showed higher community richness and diversity than fungi; additionally, opposite trends in alpha diversity were observed between bacteria and fungi over time.

#### Beta diversity analysis

**FIGURE 6:**
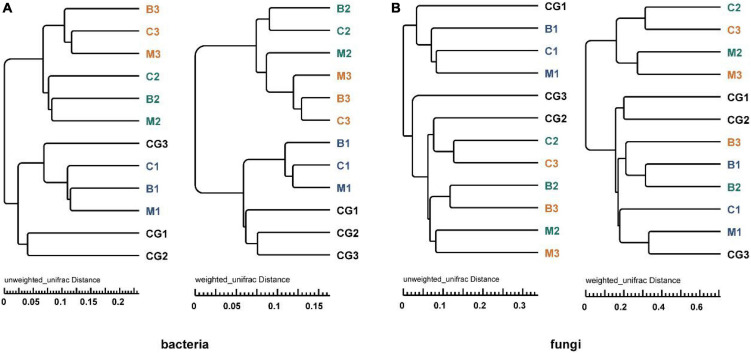
UPGMA tree analysis of bacteria and fungi based on unweighted and weighted UniFrac distance. **(A)** Bacterial community, **(B)** fungal community. The letters in group ID represents nest material composition (B, bamboo leaf; C, couch grass; M, mixed litter; CG, control group); Arabic numerals represent different incubation periods (1, pre-incubation period; 2, mid-incubation period; 3, post-incubation period).

According to the UPGMA tree analysis based on unweighted and weighted UniFrac distances, bacterial groups from the same incubation period clustered into the same cluster, especially when species richness is taken into account (Figure 6A). For fungi, groups from pre-incubation period clustered into the same cluster, and that from mid- and post-incubation period clustered into a large cluster, in this which (the large cluster) the same nest material composition clustered together. When species richness was considered, the same nest materials clustered together more obviously (Figure 6B). Variations in microbial communities are further supported by the ANOSIM (Bray–Curtis distance) and Wilcoxon test results based on weighted and unweighted UniFrac distances (*p* < 0.05) (Supplementary Table 6).

Therefore, microbial community structure was influenced by incubation period and nest material composition. The bacterial community had a stronger response to changes in incubation period than nest material, while the fungal community showed the opposite trend.

### Functional inference from taxonomy

**FIGURE 7:**
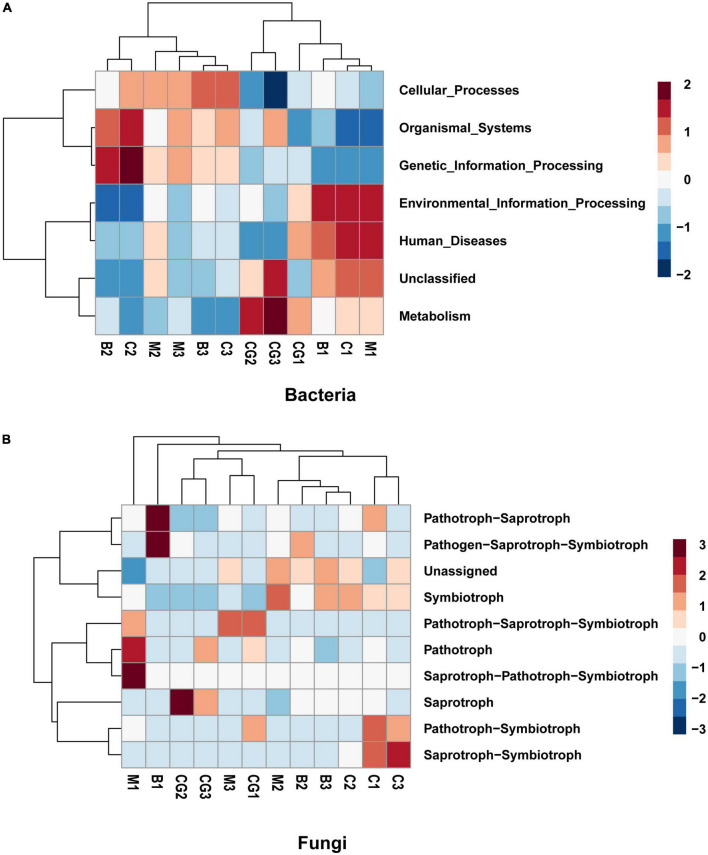
Predicted function of the bacterial and fungal communities. **(A)** Bacterial community, **(B)** fungal community. The letters in group ID represents nest material type (B, bamboo leaf; C, couch grass; M, mixed litters; CG, control group); Arabic numerals represent different incubation periods (1, pre-incubation; 2, mid-incubation; 3, post-incubation).

The study investigated the functional capacity of microbiota according to incubation period and nest material composition. Tax4Fun, an R program package based on 16S Silva database for functional prediction of gut, soil and other environmental samples, was used to predict the bacterial function. Six metabolic pathways were identified to be significant difference in the bacterial communities between the incubation periods. A shift was observed in bacterial function from pathways involved in metabolism, human disease, and environmental information processing during pre-incubation period to genetic information processing, organismal systems, and cellular processes during mid- and post-incubation period (Figure 7A).

Using the FUNGuild database, 10 ecological guilds were identified for the fungal communities, including saprotroph-saprotroph-symbiotroph, pathotroph, pathotroph-saprotroph, pathotroph-symbiotroph, saprotroph-symbiotroph, pathogen- saprotroph-symbiotroph, symbiotroph, saprotroph-pathotroph-symbiotroph, and unassigned (Figure 7B). The dominant trophic mode of Ascomycota was saprotrophic and, to a lesser degree, symbiotrophic. The dominant trophic mode among Basidiomycota varied to a greater degree. Chytridiomycota was dominated by pathotrophs, pathotroph-saprotrophs and, to a lesser degree, saprotrophs. The only trophic mode utilized by Glomeromycota is the symbiotrophic mode (Ogwu et al., 2019).

## Discussion

### Composition of microbial community on nest material

This study examined the microbial communities of Chinese alligator nests, including bacteria and fungi. The major bacterial and fungal phyla were similar in different nest material groups. Proteobacteria, Actinobacteria, Bacteroidetes, Chloroflexi, Firmicutes, Myxococcota, Planctomycetes, and Verrucomicrobia were the most abundant bacterial phyla and occur commonly in soil (Guimaraes et al., 2020). Proteobacteria, which were significantly less abundant after incubation, was the largest and most diverse bacterial phylum and included many pathogenic bacteria. Proteobacteria are one of the most dominant phyla found in soil, phyllosphere, and avian nest materials (Janssen, 2006; Vorholt, 2012; Navarro-Noya et al., 2014). Actinobacteria, are common in the nests of social arthropods (Madden et al., 2013; Otani et al., 2016; Nazipi et al., 2021), such as termites (*Syntermes wheeleri*) (Guimaraes et al., 2020). Additionally, Actinobacteria are considered to be the second-most abundant bacterial phylum in the gut of higher termites and occurs primarily in those that feed on humic matter (Hervé et al., 2020). Termite mounds are a major heat source for reptile nests and generate higher temperatures than plant materials (Magnusson, 1979). In this study, Actinobacteria was the second-most abundant bacterial phylum and remained relatively stable between nest materials. We have observed many termites on nest materials at mid- and post-incubation, thus we speculate Actinobacteria is not only from nest materials, relative abundance of Actinobacteria is not declined due the number of termites increase. In particular, this phylum could be associated with antimicrobial activity and pathogen defense (Visser et al., 2012) as they can produce antimicrobial peptides (Sujada et al., 2014; Enagbonma et al., 2019).

Ascomycota was the most abundant fungal phylum, followed by Basidiomycota, both of which can degrade cellulose (Lynd et al., 2002; De Boer et al., 2005). Ascomycota exclusively decompose carbohydrates without delignification (Osono, 2007). Basidiomycota is the most ecologically significant group of fungi involved in the degradation of plant litter (Steffen et al., 2007). Some species can produce a wide variety of oxidoreductases and hydrolytic enzymes (Colpaert and Van Laere, 1996), which can degrade recalcitrant organic compounds, such as lignin (Steffen et al., 2000), in aboveground litter and humic layers (Ghosh et al., 2003). In the study, Ascomycota and Basidiomycota play the dominant roles in degradation of nest materials and relative abundance all were less significantly at mid- and post-incubation than pre-incubation with cellulose and lignin content of nest materials reduced gradually. In addition, Rozellomycota were significantly more abundant after incubation, it also was related to amounts increasing of termites and other invertebrate species, since most members were parasites of phytoplankton, Oomycota, zooplankton, and microscopic invertebrates (Sun et al., 2019). It is important to note that the classification of Rozellomycota as a fungus is controversial since they possess some but not all of the characteristic features of fungi (Quandt et al., 2017).

At the genus level, *Allorhizobium-Neorhizobium-Pararhizobium-Rhizobium* was the most abundant bacteria in soil and sludge. Members of the genus are regarded as beneficial soil bacteria promoting phosphorus and nitrogen fixation. The dominant genus of fungi was *Fusarium*, which was present in all nest material groups during incubation period and is known to produce *Fusarium* toxins. Many species of the genus have been isolated from sea turtle eggs (Sarmiento-Ramírez et al., 2010; Hoh et al., 2020), including *Fusarium falciforme* and *Fusarium keratoplasticum* (O’Donnell et al., 2008), and are responsible for fusariosis in sea turtle eggs (Smyth et al., 2019).

### Response of microbial community dynamics to nest material composition

The study compared the microbial community diversity and structure of the different nest material compositions during the same incubation period. Alpha diversity was not significantly different; however, the difference in beta diversity was significant. In particular, the fungal community had a stronger response to nest material composition than bacteria did. The species richness of fungi is much lower than that of bacteria in nest material environment, so some small changes in the structure of the fungal community was more easily detected than bacteria. According to the LEfSe analysis, most of significant difference bacteria were mainly enriched at mid-incubation, while amost significant difference fungal taxa were enriched during pre-incubation. These results showed that nest material composition was a stronger determinant of fungal than bacterial community composition, consistent with the results of a previous study (Habtewold et al., 2020). It hypothesize that the intensity of this effect could be related to the close ecological relationship between fungi and plants. Chinese alligators choose a suitable site and then use ground weeds, branches, leaves, and soil to build a mound (Grigg and Kirshner, 2015). Variation in the nest material composition due to differences in litter species and mixing ratio could likely affect bacteria and fungi communities (Wardle et al., 2003). For example, surface soils often carry more bacteria than fungi and is a common nesting material used by Chinese alligators (Chen et al., 2003). Besides soil material, the mixing of various plant litters could increase the complexity of litter composition and nutrient sources, thus establishing more diverse niches and a larger decomposer community (Zhang et al., 2020). In conclusion, microbial community structure difference in pre-incubation was resulted by plant litter species and mixing ratio. However, during incubation, environmental factors, such as pH, temperature, humidity, light, and microbial interactions, directly affect the microbial community, thus nest material composition was not main reason why fungal community structure difference in mid- and post-incubation (Rousk et al., 2010; Newsham et al., 2016; Chapman et al., 2018; Zhang et al., 2020).

### Response of microbial community dynamics to incubation periods

Microbial community diversity and structure varied significantly between incubation periods. Bacterial species diversity and richness increased, but the relative abundances of most dominant bacteria, including many pathogenic bacteria, declined after incubation. In contrast, the diversity and richness of fungal species decreased. Generally, the relatively high temperature and moisture content of nest microbiomes favor bacterial over fungal activity (Møller et al., 1999). Moreover, humidity and temperature are more constant inside than outside the nest, irrespective of erratic weather changes. In this study, the humidity of all Chinese alligator nests was more than 95% and very stable; the humidity of the air outside the nest was significantly lower than that inside the nest and fluctuated greatly.

Another reason for the difference in bacterial and fungal diversity is the decomposition of nest materials and the reduction of nest substrates. Fungi play a major role in recalcitrant organic matter degradation; fungi perform most of the cellulose and lignin degradation in soils and litter components (De Boer et al., 2005). Bacteria can use at least part of the degradation intermediates of lignin produced by fungi (Rüttimann et al., 1991). Nest materials contained many easily decomposable matrices in pre-incubation, such as fresh plant leaves, and the relative abundance of Ascomycota reached about 70% at this stage. As time went on, nest materials gradually decomposed and were reduced, resulting in a decrease in Ascomycota abundance (Osono et al., 2005; Osono, 2007), while bacteria using simple substrates and degradation intermediates were not affected (Ko and Lockwood, 1970).

Several studies have assessed the antagonistic relationship between fungi and bacteria (Bengtsson, 1992; Tsuneda and Thorn, 1995; Møller et al., 1999). Many antifungal strategies have been identified in bacteria, which can produce direct inhibitory factors, including HCN; lytic enzymes; antibiotics and volatiles (interference competition); and nutrient-sequestering factors, such as iron-chelating siderophores (substrate competition) (Whipps, 2001; Weller et al., 2002; Wheatley, 2002). Chitinase is involved in the lysis of hyphae and the inhibition of hyphal growth. The involvement of chitinase in the inhibition of fungal growth on water-agar has also been studied (De Boer et al., 1998). Additionally, chitinolytic bacteria can use living fungal hyphae as their actual growth substrate, and bacterial antibiotics might induce autolysis of fungal mycelia (Lloyd et al., 1965). In our study, *Bacillus*, a chitinolytic soil bacterium that possesses chitinase genes, demonstrated antifungal properties. Actinobacteria are the most abundant chitin-degrading bacteria in agricultural soil (De Boer et al., 1998). In addition, Myxococcota increased after incubation; this phylum includes gram-negative bacteria and micropredators in the soil ecosystem (Shimkets, 1990; Dawid, 2000; Zhang et al., 2020). Most species of Myxococcota prey on various microorganisms, including bacteria and fungi (Bull et al., 2002; Morgan et al., 2010; Ye et al., 2020). Additionally, most species of the Firmicutes genus *Herpetosiphon* have predatory functions and can digest some bacteria completely (Lewin, 1970; Wenzel and Müller, 2009).

Although bacterial alpha diversity and richness increased after incubation, further analysis indicated that 7 dominant bacterial genera, including some pathogenic bacteria, declined after incubation. An environment already colonized by certain bacteria might make it more challenging for other bacteria to establish (Dillon et al., 2005; Lozupone and Knight, 2007; Marteyn et al., 2011). Thus, a reduction in dominant bacteria could alleviate internal resource competition, promoting diversity and stability in the bacterial community. The lower relative abundance of pathogenic microbes and higher relative abundance of beneficial microbes were consistent with previous avian studies (Song et al., 2022). Research on the Eurasian Magpie *(Pica pica)* and sea turtles (*Chelonia mydas*) has shown that *Flavobacterium*, *Acinetobacter*, and *Herpetosiphon* could be pathogenic (Lee et al., 2014; Vega-Manriquez et al., 2018); these genera primarily occur in soil, which is major nest material in Chinese alligator nests (Grigg and Kirshner, 2015). *Flavobacterium* has been repeatedly isolated from cases of ulcerative stomatitis and obstructive rhinitis (Glazebrook et al., 1993) and the genus also participates in snake dystocia (Estrada et al., 2015). *Flavobacterium* and *Acinetobacter* have also been isolated from the exocrine skin glands of 23 adult American alligators (*Alligator mississippiensis*) and African dwarf crocodiles (*Osteolaemus tetraspis*) (Williams et al., 1990; Madsen, 1993) and were present in frozen captive Nile crocodile (*Crocodylus niloticus*) tail meat (Madsen, 1993). *Pantoea* is a highly diverse group whose members are found in aquatic and terrestrial environments, and some species have been linked to diseases in plants, humans, and animals (Walterson and Stavrinides, 2015). In humans *Pantoea* contribute to septic arthritis, bacteremia, septicemia, and peritonitis, among others (Flatauer and Khan, 1978; Vincent and Szabo, 1988; De Baere et al., 2004; Bergman et al., 2007; Christakis et al., 2007; Aly et al., 2008; Labianca et al., 2013). *Psychrobacter* are rare opportunistic human pathogens (Zeng et al., 2016). Although their pathogenicity has been reported in animals, it is unclear whether they are harmful to the Chinese alligator. These pathogenic bacteria had a high relative abundance in the pre-incubation period, which declined after incubation. In addition to pathogenic bacteria, fungi can penetrate the eggshell of salt-water crocodiles (*Crocodylus porosus*), for example, in which the spore size is sufficient to allow hyphae and spores to pass through and grow along minute cracks (Huchzermeyer, 2003). The danger of fungal infection is more serious in mound-nesting crocodile species because of the plant materials used for nest construction (Huchzermeyer, 2003). *Fusarium* was found in the oral fungal flora of ten American alligators (Flandry et al., 1989) and the intestinal flora of African dwarf crocodiles (Huchzermeyer, 1997, 2002). It was also isolated from shell membranes of unhatched crocodile eggs in Zimbabwe and recovered from caseous material lodged inside the trachea and bronchi of turtles with bronchopneumonia (Glazebrook et al., 1993). Additionally, functional prediction of bacteria and fungi showed the same trend: pathways related to disease or pathogens declined after incubation. Many species of the genera *Pseudonocardia* and *Actinomadura* produce antibiotics that inhibit the activity of bacteria, fungi, and tumor cells (Maskey et al., 2003; Harada et al., 2004). In the study, relative abundance of *Pseudonocardia* and *Actinomadura* are increased but are not significant, we speculate these bacteria as beneficial bacteria to regulate stability and diversity of microbial environment by preying and restraining pathogen microorganism but they do not act on eggs directly.

## Conclusion

In summary, microbial communities fluctuated during incubation. After incubation, the relative abundance of dominant bacteria decreased, the bacterial community became more diverse, and the relative abundances of fungi and pathogenic bacteria decreased. Our findings suggested that, although Chinese alligators deposit their eggs in an untended nest, the microbial environment during incubation spontaneously reached a relatively stable and beneficial state for egg incubation. Thus, an undisturbed incubation environment is very crucial, the manager can take some measures to ensure successful hatching. Firstly, managers should keep nests safe and stable from other animals and people damaging nest sites during incubation. Secondly, the manager can increase nest material species and amounts by improving plant diversity and richness or putting bamboo leaves and straw artificially. Lastly, the manager can put some germicide on natural nest materials.

The evolutionary and ecological effects of microbes in nest material function and breeding success remain largely unclear for reptile species. In the study, a large number of literature studies are used to support our views. Further studies on various reptile species are required to determine the strength of association for particular bacteria and fungi with incubation time and nest material composition. In the future study, using metagenomics to clarify the functional differences of bacterial and fungal microbial groups is helpful. Combination of traditional culture-based method and metatranscriptomics, may help to under more function of microbes. In addition, whether there are eggs in the nest may also affect the structure and function of microorganisms in the nest, setting up aseptic environment or no-egg environment control groups is taken into account.

## Data availability statement

The data presented in this study are deposited in the NCBI SRA database, accession number: PRJNA868907.

## Author contributions

S-GF and J-QL conceived, designed, and supervised the project and revised the manuscript. Q-ZY, M-YH, and LW collected the samples. Q-ZY extracted the DNA samples. Q-ZY, M-YH, and J-QL analyzed the data and drafted the manuscript. All authors read and approved the final manuscript.

## Footnotes

1
http://ccb.jhu.edu/software/FLASH/

2
http://qiime.org/scripts/split_libraries_fastq.html

3
http://www.drive5.com/uparse/

## References

[B1] Ackerman R. A. (2017). “The nest environment and the embryonic development of sea turtles,” in *The Biology of Sea Turtles*, eds Lutz P. L. Musick J. A. (Boca Raton, FL: CRC Press), 83–106.

[B2] Aly N. Y. A. Salmeen H. N. Lila R. A. A. Nagaraja P. A. (2008). Pantoea agglomerans bloodstream infection in preterm neonates. *Med. Princ. Pract.* 17 500–503. 18836282 10.1159/000151575

[B3] Bellemain E. Carlsen T. Brochmann C. Coissac E. Taberlet P. Kauserud H. (2010). ITS as an environmental DNA barcode for fungi: an in silico approach reveals potential PCR biases. *BMC Microbiol.* 10:189. 10.1186/1471-2180-10-189 20618939 PMC2909996

[B4] Bengtsson G. (1992). Interactions between fungi, bacteria and beech leaves in a stream microcosm. *Oecologia* 89 542–549. 10.1007/BF00317161 28311885

[B5] Bergman K. A. Arends J. P. Schölvinck E. H. (2007). Pantoea agglomerans septicemia in three newborn infants. *Pediatr. Infect. Dis. J.* 26 453–454. 17468662 10.1097/01.inf.0000261200.83869.92

[B6] Bergmann G. T. Bates S. T. Eilers K. G. Lauber C. L. Caporaso J. G. Walters W. A. (2011). The under-recognized dominance of Verrucomicrobia in soil bacterial communities. *Soil Biol. Biochem.* 43 1450–1455. 10.1016/j.soilbio.2011.03.012 22267877 PMC3260529

[B7] Bokulich N. A. Subramanian S. Faith J. J. Gevers D. Gordon J. I. Knight R. (2013). Quality-filtering vastly improves diversity estimates from Illumina amplicon sequencing. *Nat. Methods* 10 57–59. 10.1038/nmeth.2276 23202435 PMC3531572

[B8] Brandl H. B. Van Dongen W. F. D. Darolová A. Krištofík J. Majtan J. Hoi H. (2014). Composition of bacterial assemblages in different components of Reed warbler nests and a possible role of egg incubation in pathogen regulation. *PLoS One* 9:e114861. 10.1371/journal.pone.0114861 25493434 PMC4262450

[B9] Bruce J. Drysdale E. M. (1994). “Trans-shell transmission,” in *Microbiology of the Avian Egg*, eds Board R. G. Fuller R. (Boston, MA: Springer), 63–91.

[B10] Bull C. T. Shetty K. G. Subbarao K. V. (2002). Interactions between Myxobacteria, plant pathogenic fungi, and biocontrol agents. *Plant Dis.* 86 889–896. 10.1094/PDIS.2002.86.8.889 30818644

[B11] Candan C. Candan E. D. (2020). Bacterial diversity of the green turtle (*Chelonia mydas*) nest environment. *Sci. Total Environ.* 720:137717. 10.1016/j.scitotenv.2020.137717 32325608

[B12] Caporaso J. G. Kuczynski J. Stombaugh J. Bittinger K. Bushman F. D. Costello E. K. (2010). QIIME allows analysis of high-throughput community sequencing data. *Nat. Methods* 7 335–336. 20383131 10.1038/nmeth.f.303 PMC3156573

[B13] Chapman S. K. Newman G. S. Hart S. C. Schweitzer J. A. Koch G. W. (2018). Leaf litter mixtures alter microbial community development: mechanisms for non-additive effects in litter decomposition. *PLoS One* 8:e62671. 10.1371/journal.pone.0062671 23658639 PMC3639160

[B14] Chen B. H. Hua T. M. Wu X. B. Wang C. L. (2003). *Research on Chinese alligator.* Shanghai: Shanghai Scientific and Technological Education Publishing House.

[B15] Chen C.-Y. Chen C.-K. Chen Y.-Y. Fang A. Shaw G. T.-S. Hung C.-M. (2020). Maternal gut microbes shape the early-life assembly of gut microbiota in passerine chicks via nests. *Microbiome* 8:129. 10.1186/s40168-020-00896-9 32917256 PMC7488855

[B16] Christakis G. B. Perlorentzou S. P. Aslanidou M. Savva L. Zarkadis I. K. (2007). Bacteremia caused by *Pantoea agglomerans* and *Enterococcus faecalis* in a patient with colon cancer. *J. BUON* 12 287–290. 17600887

[B17] Clark L. Mason J. R. (1985). Use of nest material as insecticidal and anti-pathogenic agents by the European starling. *Oecologia* 67 169–176. 10.1007/BF00384280 28311305

[B18] Coenen-Strass D. Schaarschmidt B. Lamprecht I. (1980). Temperature distribution and calorimetric determination of heat production in the nest of the Wood ant, Formica Polyctena (Hymenoptera, Formicidae). *Ecology* 61 238–244.

[B19] Colpaert J. V. Van Laere A. (1996). A comparison of the extracellular enzyme activities of two ectomycorrhizal and a leaf-saprotrophic basidiomycete colonizing beech leaf litter. *New Phytol.* 134 133–141.

[B20] Cook M. I. Beissinger S. R. Toranzos G. A. Arendt W. J. (2005a). Incubation reduces microbial growth on eggshells and the opportunity for trans-shell infection. *Ecol. Lett.* 8 532–537. 10.1111/j.1461-0248.2005.00748.x 21352457

[B21] Cook M. I. Beissinger S. R. Toranzos G. A. Rodriguez R. A. Arendt W. J. (2005b). Microbial infection affects egg viability and incubation behavior in a tropical passerine. *Behav. Ecol.* 16 30–36.

[B22] Cook M. I. Beissinger S. R. Toranzos G. A. Rodriguez R. A. Arendt W. J. (2003). Trans-shell infection by pathogenic micro-organisms reduces the shelf life of non-incubated bird’s eggs: a constraint on the onset of incubation? *Proc. R. Soc. B Biol. Sci.* 270 2233–2240. 10.1098/rspb.2003.2508 14613609 PMC1691504

[B23] Dawid W. (2000). Biology and global distribution of myxobacteria in soils. *FEMS Microbiol. Rev.* 24 403–427. 10978544 10.1111/j.1574-6976.2000.tb00548.x

[B24] De Baere T. Verhelst R. Labit C. Verschraegen G. Wauters G. Claeys G. (2004). Bacteremic infection with Pantoea ananatis. *J. Clin. Microbiol.* 42 4393–4395. 10.1128/JCM.42.9.4393-4395.2004 15365053 PMC516322

[B25] De Boer W. Folman L. B. Summerbell R. C. Boddy L. (2005). Living in a fungal world: impact of fungi on soil bacterial niche development. *FEMS Microbiol. Rev.* 29 795–811. 10.1016/j.femsre.2004.11.005 16102603

[B26] De Boer W. Klein Gunnewiek P. J. A. Lafeber P. Janse J. D. Spit B. E. Woldendorp J. W. (1998). Anti-fungal properties of chitinolytic dune soil bacteria. *Soil Biol. Biochem.* 30 193–203.

[B27] Dillon R. J. Vennard C. T. Buckling A. Charnley A. K. (2005). Diversity of locust gut bacteria protects against pathogen invasion. *Ecol. Lett.* 8 1291–1298.

[B28] Edgar R. C. (2013). UPARSE: highly accurate OTU sequences from microbial amplicon reads. *Nat. Methods* 10 996–998. 10.1038/nmeth.2604 23955772

[B29] Enagbonma B. J. Aremu B. R. Babalola O. O. (2019). Profiling the functional diversity of termite mound soil bacteria as revealed by shotgun sequencing. *Genes* 10 637. 10.3390/genes10090637 PMC6770954 31450818

[B30] Estrada D. M. Mathes K. Martínez P. P. (2015). Distocia en una serpiente ratonera amarilla (*Coelognathus flavolineatus*, Schlegel 1837) - Reporte de caso. *Rev. Facultad Med. Vet. Zootecnia* 62 75–92.

[B31] Flandry F. Lisecki E. J. Domingue G. J. Nichols R. L. Greer D. L. Haddad R. J. (1989). Initial antibiotic therapy for *Alligator* bites: characterization of the oral flora of *Alligator mississippiensis*. *South Med. J.* 82 262–266. 10.1097/00007611-198902000-00027 2783788

[B32] Flatauer F. E. Khan M. A. (1978). Septic arthritis caused by *Enterobacter agglomerans*. *Arch. Intern. Med.* 138:788. 646543

[B33] Ghosh A. Frankland J. C. Thurston C. F. Robinson C. H. (2003). Enzyme production by *Mycena galopus* mycelium in artificial media and in *Picea sitchensis* F-1 horizon needle litter. *Mycol. Res.* 107 996–1008. 10.1017/s0953756203008177 14531622

[B34] Glazebrook J. S. Campbell R. S. F. Thomas A. D. (1993). Studies on an ulcerative stomatitis obstructive rhinitis pneumonia disease complex in hatching and juvenile sea-turtles *Chelonia mydas* and *Caretta caretta*. *Dis. Aquat. Organ.* 16 133–147.

[B35] Grigg G. Kirshner D. (2015). *Biology and Evolution of Crocodylians.* London: CSIRO.

[B36] Grizard S. Dini-Andreote F. Tieleman B. I. Salles J. F. (2014). Dynamics of bacterial and fungal communities associated with eggshells during incubation. *Ecol. Evol.* 4 1140–1157. 10.1002/ece3.1011 24772289 PMC3997328

[B37] Grizard S. Versteegh M. A. Ndithia H. K. Salles J. F. Tieleman B. I. (2015). Shifts in bacterial communities of eggshells and antimicrobial activities in eggs during incubation in a ground-nesting passerine. *PLoS One* 10:e0121716. 10.1371/journal.pone.0121716 25880684 PMC4400097

[B38] Guimaraes H. I. P. Santana R. H. Silveira R. Bezerra Pinto O. H. Quirino B. F. Barreto C. C. (2020). Seasonal variations in soil microbiota profile of termite (*Syntermes wheeleri*) mounds in the Brazilian tropical savanna. *Microorganisms* 8:1482. 10.3390/microorganisms8101482 32992494 PMC7600031

[B39] Gwinner H. Berger S. (2005). European starlings: nestling condition, parasites and green nest material during the breeding season. *J. Ornithol.* 146 365–371.

[B40] Haas B. J. Gevers D. Earl A. M. Feldgarden M. Ward D. V. Giannoukos G. (2011). Chimeric 16S rRNA sequence formation and detection in Sanger and 454-pyrosequenced PCR amplicons. *Genome Res.* 21 494–504. 10.1101/gr.112730.110 21212162 PMC3044863

[B41] Habtewold J. Z. Helgason B. L. Yanni S. F. Janzen H. H. Ellert B. H. Gregorich E. G. (2020). Litter composition has stronger influence on the structure of soil fungal than bacterial communities. *Eur. J. Soil Biol.* 98:103190.

[B42] Hanmer H. J. Thomas R. L. Beswick G. J. F. Collins B. P. Fellowes M. D. E. (2017). Use of anthropogenic material affects bird nest arthropod community structure: influence of urbanisation, and consequences for ectoparasites and fledging success. *J. Ornithol.* 158 1045–1059.

[B43] Harada K.-I. Tomita K. Fujii K. Masuda K. Mikami Y. Yazawa K. (2004). Isolation and structural characterization of siderophores, madurastatins, produced by a pathogenic *Actinomadura madurae*. *J. Antibiot.* 57 125–135. 10.7164/antibiotics.57.125 15112961

[B44] Hervé V. Liu P. Dietrich C. Sillam-Dussès D. Stiblik P. Šobotnik J. (2020). Phylogenomic analysis of 589 metagenome-assembled genomes encompassing all major prokaryotic lineages from the gut of higher termites. *PeerJ* 8:e8614. 10.7717/peerj.8614 32095380 PMC7024585

[B45] Hoh D. Z. Lin Y.-F. Liu W.-A. Sidique S. N. M. Tsai I. J. (2020). Nest microbiota and pathogen abundance in sea turtle hatcheries. *Fungal Ecol.* 47:100964.

[B46] Hoi H. Schleicher B. Valera F. (1994). Female mate choice and nest desertion in penduline tits, *Remiz pendulinus*: the importance of nest quality. *Anim. Behav.* 48 743–746.

[B47] Hoi H. Schleicher B. Valera F. (1996). Nest size variation and its importance for mate choice in penduline tits, *Remiz pendulinus*. *Anim. Behav.* 51 464–466.

[B48] Houston C. S. Saunders J. R. Crawford R. D. (1997). Aerobic bacterial flora of addled raptor eggs in Saskatchewan. *J. Wildlife Dis.* 33 328–331. 10.7589/0090-3558-33.2.328 9131569

[B49] Huchzermeyer F. W. (1997). *Diseases of Farmed Crocodiles.* Cham: Springer, 169–175.

[B50] Huchzermeyer F. W. (2002). Diseases of farmed crocodiles and ostriches. *Rev. Sci. Tech. Off. Int. Epiz.* 21 265–276. 10.20506/rst.21.2.1334 11974614

[B51] Huchzermeyer F. W. (2003). *Crocodiles: Biology, Husbandry and Diseases.* Cambridge, MA: CABI Publishing.

[B52] Janssen P. H. (2006). Identifying the dominant soil bacterial taxa in libraries of 16S rRNA and 16S rRNA genes. *Appl. Environ. Microbiol.* 72 1719–1728. 16517615 10.1128/AEM.72.3.1719-1728.2006 PMC1393246

[B53] Ko W.-H. Lockwood J. L. (1970). Mechanism of lysis of fungal mycelia in soil. *Phytopathology* 60 148–154.

[B54] Kozlowski S. Malyszko E. Pinowski J. Kruszewicz A. (1991). “The effect of microorganisms on the mortality of house sparrow (*Passer domesticus*) and tree sparrow (*Passer montanus*) embryos,” in *Nestling Mortality of Granivorous Birds Due to Microorganisms and Toxic Substances*, eds Pinowski J. Kavanagh B. P. Górski W. (London: PWN), 121–128.

[B55] Labianca L. Montanaro A. Turturro F. Calderaro C. Ferretti A. (2013). Osteomyelitis caused by *Pantoea agglomerans* in a closed fracture in a child. *Orthopedics* 36 E252–E256. 23383681 10.3928/01477447-20130122-32

[B56] Lang J. W. Andrews H. V. (1994). Temperature-dependent sex determination in crocodilians. *J. Exp. Zool.* 270 28–44.

[B57] Lee W. Y. Kim M. Jablonski P. G. Choe J. C. Lee S. (2014). Effect of incubation on bacterial communities of eggshells in a temperate bird, the Eurasian magpie (*Pica pica*). *PLoS One* 9:e103959. 10.1371/journal.pone.0103959 25089821 PMC4121233

[B58] Lewin R. A. (1970). New Herpetosiphon species (Flexibacterales). *Can. J. Microbiol.* 16:517. 10.1139/m70-087 5423287

[B59] Lloyd A. B. Noveroske R. L. Lockwood J. L. (1965). Lysis of fungal mycelium by *Streptomyces* spp and their chitinase systems. *Phytopathology* 55 871–875. 5827065

[B60] López-Luna M. A. Hidalgo-Mihart M. G. Aguirre-León G. González-Ramón M. del C. Rangel-Mendoza J. A. (2015). Effect of nesting environment on incubation temperature and hatching success of Morelet’s crocodile (*Crocodylus moreletii*) in an urban lake of Southeastern Mexico. *J. Therm. Biol.* 49-50 66–73. 10.1016/j.jtherbio.2015.01.006 25774028

[B61] Lozupone C. A. Knight R. (2007). Global patterns in bacterial diversity. *Proc. Natl. Acad. Sci. U.S.A.* 104 11436–11440. 17592124 10.1073/pnas.0611525104 PMC2040916

[B62] Lynd L. R. Weimer P. J. Van Zyl W. H. Pretorius I. S. (2002). Microbial cellulose utilization: fundamentals and biotechnology. *Microbiol. Mol. Biol. Rev.* 66 506–577. 12209002 10.1128/MMBR.66.3.506-577.2002 PMC120791

[B63] Madden A. A. Grassetti A. Soriano J.-A. N. Starks P. T. (2013). Actinomycetes with antimicrobial activity isolated from Paper wasp (Hymenoptera: Vespidae: Polistinae) nests. *Environ. Entomol.* 42 703–710. 10.1603/EN12159 23905732

[B64] Madsen M. (1993). Microbial flora of frozen tail meat from captive Nile crocodiles (*Crocodylus niloticus*). *Int. J. Food Microbiol.* 18 71–76. 10.1016/0168-1605(93)90009-6 8466815

[B65] Magnusson W. E. (1979). Maintenance of temperature of crocodile nests (Reptilia, Crocodilidae). *J. Herpetol.* 13 439–443.

[B66] Magnusson W. E. Lima A. P. Sampaio R. M. (1985). Sources of heat for nests of *Paleosuchus trigonatus* and a review of crocodilian nest temperatures. *J. Herpetol.* 19 199–207.

[B67] Magoč T. Salzberg S. L. (2011). FLASH: fast length adjustment of short reads to improve genome assemblies. *Bioinformatics* 27 2957–2963. 10.1093/bioinformatics/btr507 21903629 PMC3198573

[B68] Marteyn B. Scorza F. B. Sansonetti P. J. Tang C. (2011). Breathing life into pathogens: the influence of oxygen on bacterial virulence and host responses in the gastrointestinal tract. *Cell Microbiol.* 13 171–176. 21166974 10.1111/j.1462-5822.2010.01549.x

[B69] Martínez-García Á Martín-Vivaldi M. Rodríguez-Ruano S. M. Peralta-Sánchez J. M. Valdivia E. Soler J. J. (2016). Nest bacterial environment affects microbiome of Hoopoe eggshells, but not that of the uropygial secretion. *PLoS One* 11:e0158158. 10.1371/journal.pone.0158158 27409772 PMC4943718

[B70] Maskey R. P. Li F. C. Qin S. Fiebig H. H. Laatsch H. (2003). Chandrananimycins AapproxC: production of novel anticancer antibiotics from a marine *Actinomadura* sp. Isolate M048 by variation of medium composition and growth conditions. *J. Antibiot.* 56 622–629. 10.7164/antibiotics.56.622 14513905

[B71] Mitchell T. S. Maciel J. A. Janzen F. J. (2013). Does sex-ratio selection influence nest-site choice in a reptile with temperature-dependent sex determination? *Proc. Biol. Sci.* 280:2460. 10.1098/rspb.2013.2460 24266033 PMC3813350

[B72] Møller J. Miller M. Kjøller A. (1999). Fungal–bacterial interaction on beech leaves: influence on decomposition and dissolved organic carbon quality. *Soil Biol. Biochem.* 31 367–374.

[B73] Morgan A. D. MacLean R. C. Hillesland K. L. Velicer G. J. (2010). Comparative analysis of myxococcus predation on soil bacteria. *Appl. Environ. Microbiol.* 76 6920–6927. 20802074 10.1128/AEM.00414-10 PMC2953020

[B74] Navarro-Noya Y. E. Jiménez-Aguilar A. Valenzuela-Encinas C. Alcántara-Hernández R. J. Ruíz-Valdiviezo V. M. Ponce-Mendoza A. (2014). Bacterial communities in soil under moss and lichen-moss crusts. *Geomicrobiol. J.* 31 152–160.

[B75] Nazipi S. Elberg C. L. Busck M. M. Lund M. B. Bilde T. Schramm A. (2021). The bacterial and fungal nest microbiomes in populations of the social spider *Stegodyphus dumicola*. *Syst. Appl. Microbiol.* 44:126222. 10.1016/j.syapm.2021.126222 34146923

[B76] Newsham K. K. Hopkins D. W. Carvalhais L. C. Fretwell P. T. Rushton S. P. O’Donnell A. G. (2016). Relationship between soil fungal diversity and temperature in the maritime Antarctic. *Nat. Clim. Change* 6 182–186. 10.3389/fmicb.2020.615659 33574801 PMC7870798

[B77] Noble D. W. A. Stenhouse V. Schwanz L. E. (2018). Developmental temperatures and phenotypic plasticity in reptiles: a systematic review and meta-analysis. *Biol. Rev. Camb. Philos. Soc.* 93 72–97. 28464349 10.1111/brv.12333

[B78] O’Donnell K. Sutton D. A. Fothergill A. McCarthy D. Rinaldi M. G. Brandt M. E. (2008). Molecular phylogenetic diversity, multilocus haplotype nomenclature, and in vitro antifungal resistance within the *Fusarium solani* species complex. *J. Clin. Microbiol.* 46 2477–2490. 10.1128/JCM.02371-07 18524963 PMC2519483

[B79] Ogwu M. C. Takahashi K. Dong K. Song H.-K. Moroenyane I. Waldman B. (2019). Fungal elevational Rapoport pattern from a high mountain in Japan. *Sci. Rep.* 9:6570. 10.1038/s41598-019-43025-9 31024040 PMC6484014

[B80] Osono T. (2007). Ecology of ligninolytic fungi associated with leaf litter decomposition. *Ecol. Res.* 22 955–974. 10.1139/w06-023 16917528

[B81] Osono T. Hobara S. Koba K. Kameda K. Takeda H. (2005). Immobilization of avian excreta-derived nutrients and reduced lignin decomposition in needle and twig litter in a temperate coniferous forest. *Soil Biol. Biochem.* 38 517–525.

[B82] Otani S. Hansen L. H. Sørensen S. J. Poulsen M. (2016). Bacterial communities in termite fungus combs are comprised of consistent gut deposits and contributions from the environment. *Microb. Ecol.* 71 207–220. 10.1007/s00248-015-0692-6 26518432 PMC4686563

[B83] Potter B. A. Carlson B. M. Adams A. E. Voss M. A. Vasseur J.-L. (2013). An assessment of the microbial diversity present on the surface of naturally incubated House wren eggs. *Open Ornithol. J.* 6 32–39.

[B84] Quandt C. A. Beaudet D. Corsaro D. Walochnik J. Michel R. Corradi N. (2017). The genome of an intranuclear parasite, Paramicrosporidium saccamoebae, reveals alternative adaptations to obligate intracellular parasitism. *eLife* 6:e29594. 10.7554/eLife.29594 29171834 PMC5701793

[B85] Quast C. Pruesse E. Yilmaz P. Gerken J. Schweer T. Yarza P. (2013). The SILVA ribosomal RNA gene database project: improved data processing and web-based tools. *Nucleic Acids Res.* 41 D590–D596. 10.1093/nar/gks1219 23193283 PMC3531112

[B86] Rognes T. Flouri T. Nichols B. Quince C. Mahé F. (2016). VSEARCH: a versatile open source tool for metagenomics. *PeerJ* 4:e2584. 10.7717/peerj.2584 27781170 PMC5075697

[B87] Rousk J. Bååth E. Brookes P. C. Lauber C. L. Lozupone C. Caporaso J. G. (2010). Soil bacterial and fungal communities across a pH gradient in an arable soil. *ISME J.* 4 1340–1351. 10.1038/ismej.2010.58 20445636

[B88] Ruiz-Castellano C. Ruiz-Rodriíguez M. Tomás G. Soler J. J. (2019). Antimicrobial activity of nest-lining feathers is enhanced by breeding activity in avian nests. *FEMS Microbiol. Ecol.* 95:fiz052. 10.1093/femsec/fiz052 30985888

[B89] Ruiz-Castellano C. Tomás G. Ruiz-Rodríguez M. Martín-Gálvez D. Soler J. J. (2016). Nest material shapes eggs bacterial environment. *PLoS One* 11:e0148894. 10.1371/journal.pone.0148894 26871451 PMC4752222

[B90] Rüttimann C. Vicuña R. Mozuch M. D. Kirk T. K. (1991). Limited bacterial mineralization of fungal degradation intermediates from synthetic lignin. *Appl. Environ. Microbiol.* 57 3652–3655. 10.1128/aem.57.12.3652-3655.1991 1785937 PMC184029

[B91] Sarmiento-Ramírez J. M. Abella E. Martín M. P. Tellería M. T. López-Jurado L. F. Marco A. (2010). Fusarium solani is responsible for mass mortalities in nests of loggerhead sea turtle, *Caretta caretta*, in Boavista, Cape Verde. *FEMS Microbiol. Lett.* 312 192–200. 10.1111/j.1574-6968.2010.02116.x 20875054

[B92] Segata N. Izard J. Waldron L. Gevers D. Miropolsky L. Garrett W. S. (2011). Metagenomic biomarker discovery and explanation. *Genome Biol.* 12:R60. 10.1186/gb-2011-12-6-r60 PMC3218848 21702898

[B93] Shimkets L. J. (1990). Social and developmental biology of the myxobacteria. *Microbiol. Rev.* 54 473–501. 1708086 10.1128/mr.54.4.473-501.1990 PMC372790

[B94] Smyth C. W. Sarmiento-Ramírez J. M. Short D. Diéguez-Uribeondo J. O’Donnell K. Geiser D. M. (2019). Unraveling the ecology and epidemiology of an emerging fungal disease, sea turtle egg fusariosis (STEF). *PLoS Pathog.* 15:e1007682. 10.1371/journal.ppat.1007682 31095638 PMC6521983

[B95] Song H. Lee K. Hwang I. Yang E. Ha J. Kim W. (2022). Dynamics of bacterial communities on eggshells and on nest materials during incubation in the Oriental tit (*Parus minor*). *Microb. Ecol.* [Epub ahead of print]. 10.1007/s00248-021-01927-0 35094098

[B96] Steffen K. T. Cajthaml T. Šnajdr J. Baldrian P. (2007). Differential degradation of oak (*Quercus petraea*) leaf litter by litter-decomposing basidiomycetes. *Res. Microbiol.* 158 447–455. 10.1016/j.resmic.2007.04.002 17537615

[B97] Steffen K. T. Hofrichter M. Hatakka A. (2000). Mineralisation of 14C-labelled synthetic lignin and ligninolytic enzyme activities of litter-decomposing basidiomycetous fungi. *Appl. Microbiol. Biotechnol.* 54 819–825. 10.1007/s002530000473 11152075

[B98] Suárez-Rodríguez M. López-Rull I. Garcia C. M. (2013). Incorporation of cigarette butts into nests reduces nest ectoparasite load in urban birds: new ingredients for an old recipe? *Biol. Lett.* 9:931. 10.1098/rsbl.2012.0931 23221874 PMC3565511

[B99] Sujada N. Sungthong R. Lumyong S. (2014). Termite nests as an abundant source of cultivable Actinobacteria for biotechnological purposes. *Microbes Environ.* 29 211–219. 10.1264/jsme2.me13183 24909709 PMC4103528

[B100] Sun J.-Z. Liu X.-Z. McKenzie E. H. C. Jeewon R. Liu J. K. Zhang X.-L. (2019). Fungicolous fungi: terminology, diversity, distribution, evolution, and species checklist. *Fungal Divers.* 95 337–430.

[B101] Tansey M. R. (1973). Isolation of thermophilic fungi from *Alligator* nesting material. *Mycologia* 65 594–601. 4579987

[B102] Tsuneda A. Thorn R. G. (1995). Interactions of wood decay fungi with other microorganisms, with emphasis on the degradation of cell walls. *Can. J. Bot.* 73 S1325–S1333.

[B103] Vega-Manriquez D. X. Dávila-Arrellano R. P. Eslava-Campos C. A. Jiménez E. S. Negrete-Philippe A. C. Raigoza-Figueras R. (2018). Identification of bacteria present in ulcerative stomatitis lesions of captive sea turtles *Chelonia mydas*. *Vet. Res. Commun.* 42 251–254. 10.1007/s11259-018-9728-y 29934703

[B104] Vincent K. Szabo R. M. (1988). *Enterobacter* agglomerans osteomyelitits of the hand from a rose thorn: a case report. *Orthopedics* 11 465–467. 10.3928/0147-7447-19880301-11 3368414

[B105] Visser A. A. Nobre T. Currie C. R. Aanen D. K. Poulsen M. (2012). Exploring the potential for Actinobacteria as defensive symbionts in fungus-growing termites. *Microb. Ecol.* 63 975–985. 10.1007/s00248-011-9987-4 22173371

[B106] Vorholt J. A. (2012). Microbial life in the phyllosphere. *Nat. Rev. Microbiol.* 10 828–840. 23154261 10.1038/nrmicro2910

[B107] Walterson A. M. Stavrinides J. (2015). Pantoea: insights into a highly versatile and diverse genus within the *Enterobacteriaceae*. *FEMS Microbiol. Rev.* 39 968–984. 10.1093/femsre/fuv027 26109597

[B108] Wardle D. A. Nilsson M.-C. Zackrisson O. Gallet C. (2003). Determinants of litter mixing effects in a Swedish boreal forest. *Soil Biol. Biochem.* 35 827–835.

[B109] Weidinger K. (2002). Interactive effects of concealment, parental behaviour and predators on the survival of open passerine nests. *J. Anim. Ecol.* 71 424–437.

[B110] Weller D. M. Raaijmakers J. M. Gardener B. B. Thomashow L. S. (2002). Microbial populations responsible for specific soil suppressiveness to plant pathogens. *Annu. Rev. Phytopathol.* 40 309–348. 10.1146/annurev.phyto.40.030402.110010 12147763

[B111] Wenzel S. C. Müller R. (2009). The impact of genomics on the exploitation of the myxobacterial secondary metabolome. *Nat. Prod. Rep.* 26 1385–1407. 10.1039/b817073h 19844638

[B112] Wheatley R. E. (2002). The consequences of volatile organic compound mediated bacterial and fungal interactions. *Antonie Van Leeuwenhoek* 81 357–364. 12448734 10.1023/a:1020592802234

[B113] Whipps J. M. (2001). Microbial interactions and biocontrol in the rhizosphere. *J. Exp. Bot.* 52 487–511. 11326055 10.1093/jexbot/52.suppl_1.487

[B114] Williams P. A. Mitchell W. Wilson G. R. Weldon P. J. (1990). Bacteria in the gular and paracloacal glands of the American alligator (*Alligator mississippiensis*; Reptila, Crocodilia). *Lett. Appl. Microbiol.* 10 73–76.

[B115] Ye X. Li Z. Luo X. Wang W. Li Y. Li R. (2020). A predatory myxobacterium controls cucumber *Fusarium* wilt by regulating the soil microbial community. *Microbiome* 8:49. 10.1186/s40168-020-00824-x 32252828 PMC7137222

[B116] Zeng Y.-X. Yu Y. Liu Y. Li H.-R. (2016). Psychrobacter glaciei sp. nov., isolated from the ice core of an Arctic glacier. *Int. J. Syst. Evol. Microbiol.* 66 1792–1798. 10.1099/ijsem.0.000939 26827927

[B117] Zhang Y. Li X. Zhang D. Qin Y. Zhou Y. Song S. (2020). Characteristics of fungal community structure during the decomposition of mixed foliage litter from *Pinus massoniana* and broadleaved tree species in southwestern China. *Chin. J. Plant Ecol.* 13 574–588.

[B118] Zhao L. Yang H.-Q. Fang L.-M. Pan G.-L. Zou W.-Q. Ren D. B. (2013). The sex ratio of wild Chinese alligators *Alligator sinensis*. *Curr. Zool.* 59 725–731.

