# Supplementary material for: Incubation determines favorable microbial communities in Chinese alligator nests
Source: Front Microbiol. 2022 Oct 13;13:983808. doi: 10.3389/fmicb.2022.983808 (PMC9606745; doi:10.3389/fmicb.2022.983808)
Supplement: Supplementary file 1 [file Table_1.DOCX]

| Date | Sample Name | Nest Material Composition | Mean  Temperature (℃) | Group  Name | incubation  Period |
| --- | --- | --- | --- | --- | --- |
| 2021/6/25-6/27 | A02, A04, A05, A06, A07, A08, A14, A15 | bamboo leaves, soil | 27.37 | B1 | pre-incubation |
|  | A09, A10, A11, A12, A16, A17, A18, A20, A21 | couch grass, other herbaceous, soil |  | C1 |  |
|  | A01, A03, A13, A19, A22, A23, A24, A25, A26, A27, A28, A29, A30 | mixed plant litter and soil, including leaves and branches of some woody plants, such as camphor and osmanthus |  | M1 |  |
|  | A42, A43, A44, A45 | plant litter and surface soil samples were collected from the area surrounding the nests |  | CG1 |  |
| 2021/7/28-7/30 | B02, B04, B05, B06, B07, B08, B14, B15 | bamboo leaves, soil | 29.14 | B2 | mid-preincubation |
|  | B09, B10, B11, B12, B16, B17, B18, B20, B21 | couch grass, other herbaceous, soil |  | C2 |  |
|  | B01, B303, B13, B19, B22, B23, B24, B25, B26, B27, B28, B29, B30 | mixed plant litter and soil, including leaves and branches of some woody plants, such as camphor and osmanthus |  | M2 |  |
|  | B42, B43, B44, B45 | plant litter and surface soil samples were collected from the area surrounding the nests |  | CG2 |  |
| 2021/8/22-8/24 | C02, C04, C05, C06, C07, C08, C14, C15 | bamboo leaves, soil | 27.83 | B3 | post-incubation |
|  | C09, C10, C11, C12, C16, C17, C18, C20, C21 | couch grass, other herbaceous, soil |  | C3 |  |
|  | C01, C03, C13, C19, C22, C23, C24, C25, C26, C27, C28, C29, C30 | mixed plant litter and soil, including leaves and branches of some woody plants, such as camphor and osmanthus |  | M3 |  |
|  | C42, C43, C44, C45 | plant litter and surface soil samples were collected from the area surrounding the nests |  | CG3 |  |

**Table S1.** Basic information of nest materials and control group samples

The letters in group ID represents nest material composition (B, bamboo leaf; C, couch grass; M, mixed litter; CG, control group); Arabic numerals represent different incubation periods (1, pre-incubation; 2, mid-incubation; 3, post-incubation).
