# Supplementary material for: Incubation determines favorable microbial communities in Chinese alligator nests
Source: Front Microbiol. 2022 Oct 13;13:983808. doi: 10.3389/fmicb.2022.983808 (PMC9606745; doi:10.3389/fmicb.2022.983808)
Supplement: Supplementary file 2 [file Table_2.DOCX]

**Table S2.** Results of 16S rRNA gene sequencing of nest material samples and control group samples microbiota in nest of Chinese alligator

| Sample  Name | Raw  reads | Clean  reads | Effective  reads | AvgLen  (nt) | Q20 | Q30 | GC  (%) | Effective  (%) | Group  Name |
| --- | --- | --- | --- | --- | --- | --- | --- | --- | --- |
| A02 | 89703 | 89139 | 68869 | 373 | 98.71 | 95.76 | 54.49 | 76.15 | B1 |
| A04 | 84436 | 83915 | 65483 | 373 | 98.71 | 95.75 | 55.19 | 76.93 |  |
| A05 | 85884 | 85480 | 67422 | 373 | 98.70 | 95.71 | 55.02 | 78.36 |  |
| A06 | 89033 | 88502 | 68493 | 373 | 98.65 | 95.51 | 54.15 | 76.80 |  |
| A07 | 84413 | 83939 | 65683 | 373 | 98.66 | 95.54 | 54.15 | 77.20 |  |
| A08 | 80945 | 80487 | 62384 | 373 | 98.71 | 95.69 | 54.67 | 76.81 |  |
| A14 | 81220 | 80879 | 64049 | 373 | 98.68 | 95.63 | 54.24 | 78.52 |  |
| A15 | 81361 | 81050 | 63665 | 373 | 98.58 | 95.33 | 54.65 | 78.14 |  |
| B02 | 88529 | 87917 | 68120 | 373 | 98.71 | 95.75 | 55.09 | 76.30 | B2 |
| B04 | 66850 | 66306 | 59951 | 373 | 98.75 | 95.83 | 54.51 | 88.87 |  |
| B05 | 90945 | 90429 | 69891 | 374 | 98.72 | 95.73 | 54.95 | 76.83 |  |
| B06 | 86691 | 86133 | 65686 | 374 | 98.75 | 95.80 | 54.93 | 74.93 |  |
| B07 | 83362 | 82957 | 66578 | 373 | 98.68 | 95.64 | 54.25 | 79.81 |  |
| B08 | 80712 | 80302 | 63063 | 374 | 98.74 | 95.84 | 54.39 | 78.13 |  |
| B14 | 84166 | 83703 | 63730 | 373 | 98.58 | 95.36 | 55.57 | 74.96 |  |
| B15 | 80579 | 80075 | 62000 | 373 | 98.78 | 95.92 | 55.29 | 76.36 |  |
| C02 | 89325 | 88824 | 67997 | 373 | 98.74 | 95.78 | 55.72 | 75.31 | B3 |
| C04 | 80736 | 80105 | 61570 | 373 | 98.68 | 95.67 | 55.05 | 75.42 |  |
| C05 | 88919 | 88315 | 69381 | 373 | 98.74 | 95.85 | 55.21 | 77.64 |  |
| C06 | 89005 | 88333 | 69865 | 373 | 98.66 | 95.65 | 55.51 | 78.07 |  |
| C07 | 81551 | 81058 | 62604 | 373 | 98.58 | 95.45 | 55.46 | 76.55 |  |
| C08 | 88976 | 88425 | 67445 | 373 | 98.63 | 95.55 | 55.30 | 74.78 |  |
| C14 | 89375 | 88804 | 68871 | 373 | 98.69 | 95.67 | 54.97 | 76.30 |  |
| C15 | 88520 | 87780 | 69315 | 373 | 98.73 | 95.77 | 55.11 | 77.43 |  |
| A09 | 78035 | 77607 | 60444 | 373 | 98.65 | 95.53 | 54.38 | 77.19 | C1 |
| A10 | 82578 | 82179 | 64751 | 373 | 98.65 | 95.54 | 54.56 | 78.09 |  |
| A11 | 87339 | 86798 | 68234 | 373 | 98.68 | 95.59 | 53.93 | 77.28 |  |
| A12 | 82019 | 81637 | 62525 | 373 | 98.61 | 95.46 | 54.13 | 76.09 |  |
| A16 | 82363 | 81892 | 64365 | 373 | 98.73 | 95.73 | 54.53 | 77.06 |  |
| A17 | 59601 | 59250 | 50698 | 373 | 98.75 | 95.76 | 54.26 | 83.61 |  |
| A18 | 88991 | 88383 | 69462 | 373 | 98.74 | 95.79 | 54.08 | 77.35 |  |
| A20 | 82077 | 81644 | 64902 | 373 | 98.81 | 95.97 | 54.64 | 78.72 |  |
| A21 | 80220 | 79761 | 62926 | 373 | 98.75 | 95.82 | 54.66 | 78.27 |  |
| B09 | 83183 | 82725 | 63834 | 374 | 98.77 | 95.90 | 54.91 | 76.22 | C2 |
| B10 | 80184 | 79688 | 61374 | 373 | 98.68 | 95.62 | 54.72 | 75.96 |  |
| B11 | 57402 | 57074 | 51819 | 374 | 98.79 | 95.93 | 54.30 | 88.66 |  |
| B12 | 55694 | 55326 | 50096 | 373 | 98.79 | 95.95 | 54.75 | 88.07 |  |
| B16 | 78842 | 78366 | 62455 | 373 | 98.72 | 95.77 | 55.62 | 79.03 |  |
| B17 | 83679 | 83325 | 65842 | 373 | 98.45 | 95.07 | 55.52 | 78.31 |  |
| B18 | 75219 | 74987 | 62489 | 373 | 98.43 | 94.96 | 55.34 | 82.58 |  |
| B20 | 78967 | 78610 | 62189 | 373 | 98.52 | 95.24 | 55.40 | 77.94 |  |
| B21 | 90049 | 89661 | 69796 | 373 | 98.67 | 95.62 | 55.64 | 76.60 |  |
| C09 | 79708 | 79273 | 62680 | 373 | 98.35 | 94.88 | 55.76 | 77.71 | C3 |
| C10 | 79388 | 78943 | 60505 | 373 | 98.61 | 95.53 | 55.25 | 75.92 |  |
| C11 | 79828 | 79477 | 61080 | 373 | 98.70 | 95.75 | 55.34 | 76.47 |  |
| C12 | 80343 | 79846 | 61499 | 373 | 98.65 | 95.62 | 54.85 | 75.66 |  |
| C16 | 81067 | 80666 | 62746 | 373 | 98.68 | 95.65 | 55.13 | 77.30 |  |
| C17 | 87462 | 86958 | 67655 | 373 | 98.75 | 95.86 | 55.23 | 77.18 |  |
| C18 | 83411 | 82749 | 64911 | 373 | 98.67 | 95.68 | 55.53 | 76.95 |  |
| C20 | 82998 | 82530 | 64194 | 373 | 98.62 | 95.53 | 55.44 | 76.20 |  |
| C21 | 81349 | 80963 | 62141 | 373 | 98.38 | 94.94 | 55.68 | 76.09 |  |
| A01 | 86887 | 86263 | 66543 | 373 | 98.69 | 95.71 | 54.03 | 75.69 | M1 |
| A03 | 81594 | 81088 | 62943 | 373 | 98.55 | 95.31 | 54.86 | 76.53 |  |
| A13 | 77872 | 77410 | 60255 | 373 | 98.68 | 95.63 | 53.74 | 76.69 |  |
| A19 | 82547 | 82074 | 64678 | 373 | 98.70 | 95.69 | 54.19 | 77.70 |  |
| A22 | 79128 | 78727 | 65840 | 373 | 98.47 | 95.10 | 54.95 | 83.01 |  |
| A23 | 81876 | 81600 | 62428 | 373 | 98.57 | 95.36 | 54.18 | 76.20 |  |
| A24 | 82169 | 81840 | 63358 | 373 | 98.43 | 95.05 | 54.35 | 76.52 |  |
| A25 | 86272 | 85875 | 67615 | 373 | 98.70 | 95.72 | 53.77 | 78.00 |  |
| A26 | 82463 | 82143 | 64277 | 373 | 98.71 | 95.76 | 54.15 | 77.75 |  |
| A27 | 81488 | 81034 | 62689 | 373 | 98.65 | 95.56 | 54.11 | 76.11 |  |
| A28 | 89370 | 88967 | 68062 | 373 | 98.82 | 95.98 | 54.69 | 75.88 |  |
| A29 | 83606 | 83120 | 65169 | 373 | 98.78 | 95.88 | 54.54 | 77.06 |  |
| A30 | 75862 | 75443 | 60056 | 373 | 98.84 | 95.97 | 53.69 | 78.69 |  |
| B01 | 82774 | 82056 | 62511 | 373 | 98.71 | 95.74 | 53.99 | 74.43 | M2 |
| B03 | 57090 | 56643 | 51181 | 373 | 98.78 | 95.93 | 54.45 | 88.20 |  |
| B13 | 84631 | 84069 | 66368 | 373 | 98.74 | 95.80 | 54.77 | 77.62 |  |
| B19 | 79118 | 78780 | 60624 | 373 | 98.33 | 94.73 | 55.73 | 75.95 |  |
| B22 | 84529 | 84127 | 63694 | 373 | 98.56 | 95.32 | 55.45 | 74.52 |  |
| B23 | 89381 | 88959 | 69826 | 373 | 98.59 | 95.41 | 54.55 | 77.47 |  |
| B24 | 80991 | 80665 | 61617 | 373 | 98.31 | 94.78 | 54.57 | 75.10 |  |
| B25 | 81835 | 81385 | 61970 | 373 | 98.59 | 95.44 | 54.18 | 74.53 |  |
| B26 | 88624 | 88275 | 69655 | 373 | 98.75 | 95.85 | 54.87 | 77.95 |  |
| B27 | 84047 | 83760 | 64229 | 373 | 98.62 | 95.48 | 54.53 | 76.40 |  |
| B28 | 79317 | 78992 | 60339 | 373 | 98.82 | 96.03 | 54.58 | 75.65 |  |
| B29 | 82890 | 82469 | 64435 | 373 | 98.81 | 95.96 | 54.55 | 77.03 |  |
| B30 | 80897 | 80378 | 61688 | 373 | 98.63 | 95.54 | 55.16 | 75.67 |  |
| C01 | 80511 | 80201 | 63284 | 373 | 98.78 | 95.93 | 55.18 | 78.57 | M3 |
| C03 | 79496 | 78943 | 60728 | 373 | 98.69 | 95.70 | 54.74 | 76.03 |  |
| C13 | 79483 | 79003 | 62310 | 373 | 98.70 | 95.74 | 55.17 | 77.36 |  |
| C19 | 86933 | 86466 | 66467 | 373 | 98.61 | 95.50 | 55.70 | 76.19 |  |
| C22 | 83289 | 82881 | 66247 | 373 | 98.65 | 95.62 | 55.47 | 79.17 |  |
| C23 | 82526 | 82167 | 63442 | 373 | 98.73 | 95.79 | 55.83 | 76.35 |  |
| C24 | 86866 | 86397 | 69023 | 373 | 98.68 | 95.67 | 55.21 | 79.03 |  |
| C25 | 81979 | 81513 | 62264 | 373 | 98.75 | 95.84 | 55.34 | 75.16 |  |
| C26 | 79853 | 79460 | 63657 | 373 | 98.70 | 95.69 | 55.81 | 79.65 |  |
| C27 | 87022 | 86241 | 66416 | 374 | 98.66 | 95.62 | 56.35 | 75.28 |  |
| C28 | 82625 | 82013 | 63996 | 373 | 98.67 | 95.64 | 55.47 | 76.83 |  |
| C29 | 79292 | 78711 | 61940 | 373 | 98.72 | 95.79 | 55.52 | 77.67 |  |
| C30 | 84264 | 83505 | 63627 | 374 | 98.63 | 95.58 | 56.41 | 74.63 |  |
| A42 | 81233 | 80891 | 63885 | 373 | 98.58 | 95.38 | 54.55 | 78.56 | CG1 |
| A43 | 85914 | 85436 | 66347 | 373 | 98.79 | 95.93 | 54.30 | 76.48 |  |
| A44 | 85699 | 85170 | 67002 | 373 | 98.68 | 95.65 | 54.07 | 77.09 |  |
| A45 | 76753 | 76485 | 60145 | 373 | 98.40 | 94.93 | 54.76 | 78.32 |  |
| B42 | 89321 | 88717 | 68057 | 373 | 98.76 | 95.86 | 54.73 | 75.89 | CG2 |
| B43 | 88540 | 87892 | 66605 | 373 | 98.77 | 95.89 | 55.05 | 74.42 |  |
| B44 | 59582 | 59169 | 52563 | 373 | 98.84 | 96.11 | 54.87 | 86.89 |  |
| B45 | 77094 | 76517 | 61855 | 373 | 98.75 | 95.87 | 55.43 | 79.43 |  |
| C42 | 86715 | 86066 | 67622 | 373 | 98.66 | 95.64 | 55.15 | 77.50 | CG3 |
| C43 | 80899 | 80426 | 66250 | 373 | 98.51 | 95.29 | 55.16 | 81.77 |  |
| C44 | 86904 | 86457 | 66299 | 373 | 98.59 | 95.47 | 54.73 | 76.09 |  |
| C45 | 82199 | 81802 | 62213 | 373 | 98.37 | 94.95 | 55.40 | 75.18 |  |
| Average | 81956 | 81489 | 63941 | 373 | 98.66 | 95.61 | 54.89 | 77.64 |  |

The letters in group ID represents nest material composition (B, bamboo leaf; C, couch grass; M, mixed litter; CG, control group); Arabic numerals represent different incubation periods (1, pre-incubation; 2, mid-incubation; 3, post-incubation).
