# Supplementary material for: Incubation determines favorable microbial communities in Chinese alligator nests
Source: Front Microbiol. 2022 Oct 13;13:983808. doi: 10.3389/fmicb.2022.983808 (PMC9606745; doi:10.3389/fmicb.2022.983808)
Supplement: Supplementary file 3 [file Table_3.DOCX]

**Table S3.** Results of ITS sequencing of nest material samples and control group samples microbiota in nest of Chinese alligator

| Sample  Name | Raw  reads | Clean  reads | Effective reads | AvgLen  (nt) | Q20 | Q30 | GC  (%) | Effective  (%) | Group Name |
| --- | --- | --- | --- | --- | --- | --- | --- | --- | --- |
| A02 | 82375 | 82016 | 80839 | 216 | 99.36 | 97.98 | 44.76 | 97.74 | B1 |
| A04 | 85127 | 84683 | 82099 | 228 | 99.29 | 97.68 | 49.29 | 94.23 |  |
| A05 | 77816 | 77483 | 74900 | 220 | 99.31 | 97.77 | 47.9 | 94.36 |  |
| A06 | 85474 | 84981 | 82471 | 226 | 99.22 | 97.43 | 47.88 | 94.03 |  |
| A07 | 84804 | 84293 | 82383 | 224 | 99.28 | 97.58 | 45.77 | 96.27 |  |
| A08 | 88804 | 88310 | 86418 | 226 | 99.26 | 97.51 | 46.72 | 95.27 |  |
| A14 | 86082 | 85643 | 82701 | 224 | 99.31 | 97.76 | 48.22 | 93.98 |  |
| A15 | 84699 | 84203 | 81273 | 235 | 99.26 | 97.47 | 46.72 | 95.17 |  |
| B02 | 75638 | 75403 | 70242 | 223 | 99.42 | 98.23 | 47.69 | 92.48 | B2 |
| B04 | 79462 | 79009 | 77806 | 226 | 99.09 | 96.88 | 46.15 | 94.64 |  |
| B05 | 78453 | 78215 | 75856 | 223 | 99.51 | 98.37 | 47.52 | 94.38 |  |
| B06 | 86638 | 86399 | 85443 | 218 | 99.56 | 98.52 | 45.88 | 96.19 |  |
| B07 | 82531 | 82244 | 80349 | 222 | 99.5 | 98.36 | 47.48 | 95.02 |  |
| B08 | 77422 | 77223 | 76132 | 219 | 99.59 | 98.63 | 47.61 | 95.05 |  |
| B14 | 83128 | 82762 | 66907 | 237 | 99.3 | 97.73 | 49.74 | 77.73 |  |
| B15 | 80272 | 79919 | 75944 | 238 | 99.42 | 98.01 | 48.09 | 93.25 |  |
| C02 | 88357 | 87926 | 86976 | 243 | 99.45 | 98.01 | 41.79 | 96.09 | B3 |
| C04 | 89834 | 89220 | 86631 | 291 | 99.19 | 96.87 | 42.97 | 94.77 |  |
| C05 | 79130 | 78838 | 76750 | 235 | 99.51 | 98.25 | 47.21 | 95.3 |  |
| C06 | 82843 | 82568 | 80444 | 224 | 99.52 | 98.39 | 47.32 | 96.33 |  |
| C07 | 77230 | 76956 | 75652 | 222 | 99.48 | 98.25 | 45.65 | 95.92 |  |
| C08 | 80675 | 80226 | 79393 | 251 | 99.35 | 97.66 | 52.72 | 96.42 |  |
| C15 | 84616 | 83857 | 76875 | 259 | 99.07 | 96.86 | 46.64 | 90.18 |  |
| A09 | 89064 | 88572 | 81487 | 229 | 99.26 | 97.62 | 48.39 | 90.83 | C1 |
| A10 | 88590 | 88047 | 82652 | 240 | 99.16 | 97.19 | 49.85 | 93 |  |
| A11 | 77966 | 77553 | 62131 | 235 | 99.04 | 97.09 | 48.34 | 78.36 |  |
| A12 | 83200 | 82667 | 77114 | 233 | 99.16 | 97.2 | 48.92 | 91.95 |  |
| A16 | 85387 | 84878 | 76257 | 235 | 99.05 | 96.76 | 48.57 | 88.9 |  |
| A17 | 86574 | 86041 | 81278 | 229 | 99.28 | 97.62 | 48.97 | 90.98 |  |
| A18 | 78036 | 77445 | 68401 | 246 | 99.06 | 96.85 | 46.56 | 86.12 |  |
| A20 | 83281 | 82650 | 79313 | 234 | 98.85 | 96.89 | 49.45 | 95.08 |  |
| A21 | 65400 | 65292 | 49787 | 203 | 99.13 | 97.62 | 49.33 | 73.03 |  |
| B09 | 86576 | 86235 | 72824 | 237 | 99.44 | 98.04 | 49.48 | 83.78 | C2 |
| B10 | 87266 | 86944 | 83772 | 229 | 99.48 | 98.29 | 54.03 | 94.51 |  |
| B11 | 81278 | 80995 | 76348 | 236 | 99.46 | 98.12 | 50.35 | 93.19 |  |
| B12 | 82509 | 82123 | 77505 | 246 | 99.4 | 97.83 | 50.13 | 90.81 |  |
| B16 | 81887 | 81284 | 70820 | 290 | 99.27 | 97.19 | 47.15 | 83.87 |  |
| B17 | 78640 | 78418 | 75786 | 214 | 99.53 | 98.55 | 49.9 | 93.1 |  |
| B18 | 77970 | 77619 | 74796 | 226 | 99.44 | 98.2 | 51.28 | 95.63 |  |
| B20 | 81943 | 81666 | 66610 | 221 | 99.44 | 98.2 | 50.51 | 80.55 |  |
| B21 | 89204 | 88741 | 82077 | 233 | 99.17 | 97.09 | 52.52 | 90.22 |  |
| C09 | 79501 | 79129 | 74566 | 231 | 99.49 | 98.29 | 51.36 | 92.72 | C3 |
| C10 | 81498 | 81205 | 79620 | 226 | 99.5 | 98.23 | 45.93 | 96.93 |  |
| C11 | 78474 | 78058 | 75782 | 243 | 99.38 | 97.83 | 52.14 | 94.35 |  |
| C12 | 84858 | 84587 | 81949 | 232 | 99.52 | 98.37 | 49.1 | 94.5 |  |
| C16 | 88748 | 87734 | 86725 | 298 | 99 | 96.31 | 47.13 | 94.98 |  |
| C17 | 87905 | 87403 | 81722 | 222 | 99.24 | 97.76 | 49.72 | 91.82 |  |
| C18 | 86712 | 85904 | 83285 | 259 | 98.95 | 96.4 | 46.36 | 95.6 |  |
| C20 | 79883 | 79190 | 70467 | 258 | 99.04 | 96.79 | 48.67 | 85.71 |  |
| C21 | 77596 | 77394 | 60751 | 217 | 99.34 | 97.94 | 48.93 | 77.26 |  |
| A01 | 86083 | 85589 | 80411 | 230 | 99.08 | 97.21 | 48.23 | 90.65 | M1 |
| A03 | 47664 | 47453 | 42073 | 224 | 99.36 | 97.85 | 47.3 | 87.71 |  |
| A13 | 79542 | 79125 | 74932 | 224 | 99.27 | 97.73 | 48.3 | 93.27 |  |
| A19 | 82385 | 81803 | 79291 | 228 | 99.19 | 97.44 | 47.3 | 95.83 |  |
| A22 | 81078 | 80520 | 74699 | 241 | 99.24 | 97.4 | 46.27 | 91.04 |  |
| A23 | 81730 | 81139 | 76423 | 246 | 98.96 | 96.57 | 46.79 | 92.75 |  |
| A24 | 89104 | 88526 | 84211 | 232 | 99.27 | 97.61 | 46.24 | 93.16 |  |
| A25 | 82845 | 82483 | 65197 | 231 | 99.17 | 97.42 | 49.55 | 77.66 |  |
| A26 | 85617 | 85012 | 81100 | 236 | 99.22 | 97.38 | 47.77 | 93.8 |  |
| A27 | 85620 | 84729 | 80988 | 278 | 98.88 | 95.77 | 47.49 | 94.27 |  |
| A28 | 84633 | 84145 | 80658 | 233 | 99.25 | 97.51 | 47.44 | 93.29 |  |
| A29 | 86088 | 85683 | 68033 | 254 | 98.99 | 96.55 | 44.46 | 77.03 |  |
| A30 | 85588 | 85002 | 82291 | 242 | 99.23 | 97.38 | 46.8 | 95.15 |  |
| B01 | 79781 | 79456 | 78659 | 231 | 99.38 | 97.92 | 49.43 | 95.76 | M2 |
| B03 | 83968 | 83881 | 81310 | 190 | 99.75 | 99.16 | 47.07 | 93.62 |  |
| B13 | 79896 | 79703 | 76502 | 207 | 99.62 | 98.76 | 45.08 | 95.34 |  |
| B19 | 88089 | 87670 | 82249 | 228 | 99.42 | 98.16 | 50.28 | 90.34 |  |
| B22 | 88418 | 88050 | 81159 | 229 | 99.47 | 98.24 | 47.07 | 91.1 |  |
| B23 | 87524 | 87040 | 83991 | 213 | 99.23 | 97.83 | 46.03 | 95.63 |  |
| B24 | 80114 | 79759 | 74952 | 210 | 99.2 | 97.93 | 44.97 | 91.66 |  |
| B25 | 88302 | 87759 | 77954 | 223 | 99.17 | 97.59 | 47.33 | 87.74 |  |
| B26 | 84532 | 83882 | 73181 | 239 | 98.94 | 96.82 | 44.97 | 85.79 |  |
| B27 | 90173 | 89756 | 82453 | 215 | 99.27 | 97.97 | 49.31 | 91.36 |  |
| B28 | 79191 | 78741 | 72753 | 226 | 99.14 | 97.47 | 48.1 | 89.52 |  |
| B29 | 81253 | 80749 | 79178 | 222 | 99.22 | 97.63 | 47.12 | 94.51 |  |
| B30 | 88902 | 88264 | 85790 | 237 | 99.18 | 97.42 | 44.95 | 95.17 |  |
| C01 | 83710 | 83411 | 81663 | 222 | 99.46 | 98.26 | 44.35 | 94.66 | M3 |
| C03 | 86403 | 86235 | 82248 | 199 | 99.67 | 98.92 | 47.67 | 93.84 |  |
| C13 | 83760 | 83479 | 82980 | 217 | 99.57 | 98.64 | 49 | 97.76 |  |
| C19 | 76664 | 76099 | 71682 | 222 | 98.85 | 97.12 | 49.26 | 90.59 |  |
| C22 | 79148 | 78783 | 74294 | 218 | 99.34 | 98.04 | 44.5 | 93.27 |  |
| C23 | 81202 | 80847 | 79370 | 222 | 99.35 | 97.98 | 43.84 | 95.07 |  |
| C24 | 84259 | 83930 | 82767 | 209 | 99.37 | 98.23 | 44.29 | 95.34 |  |
| C25 | 81198 | 80628 | 78629 | 241 | 99.23 | 97.43 | 50.39 | 95.41 |  |
| C26 | 78693 | 78282 | 76801 | 224 | 99.25 | 97.76 | 51.39 | 97.21 |  |
| C27 | 83705 | 83269 | 76144 | 223 | 99.33 | 97.94 | 47.9 | 88.7 |  |
| C28 | 87458 | 86847 | 84993 | 248 | 99.11 | 97.16 | 52.96 | 96.15 |  |
| C29 | 83427 | 82955 | 81241 | 224 | 99.24 | 97.69 | 47.98 | 94.22 |  |
| C30 | 60065 | 59342 | 54185 | 316 | 98.97 | 96.13 | 47.68 | 89.98 |  |
| A42 | 86091 | 85432 | 83396 | 242 | 99.2 | 97.12 | 43.18 | 95.48 | CG1 |
| A43 | 90430 | 89715 | 86209 | 241 | 98.92 | 96.87 | 47.77 | 92.78 |  |
| A44 | 87116 | 86708 | 81922 | 232 | 99.33 | 97.79 | 46.28 | 93.2 |  |
| A45 | 86241 | 85682 | 83179 | 234 | 99.25 | 97.45 | 47.55 | 96.41 |  |
| B42 | 84664 | 84394 | 80288 | 231 | 99.51 | 98.27 | 48.43 | 94.34 | CG2 |
| B43 | 81702 | 81357 | 73220 | 239 | 99.49 | 98.23 | 45.39 | 88.08 |  |
| B44 | 87886 | 87520 | 84404 | 232 | 99.18 | 97.71 | 48.12 | 94.75 |  |
| B45 | 81180 | 80739 | 77241 | 266 | 99.21 | 97.19 | 46.5 | 93.21 |  |
| C42 | 87272 | 86779 | 85869 | 236 | 99.34 | 97.85 | 46.07 | 96.98 | CG3 |
| C43 | 86930 | 86388 | 77920 | 234 | 99.24 | 97.47 | 48.38 | 88.55 |  |
| C44 | 82631 | 82175 | 77628 | 222 | 99.24 | 97.67 | 44.41 | 91.73 |  |
| C45 | 80242 | 79724 | 76095 | 240 | 99.26 | 97.52 | 46.88 | 92.88 |  |
| Average | 82788 | 82344 | 77556 | 233 | 99 | 98 | 48 | 92 |  |

The letters in group ID represents nest material composition (B, bamboo leaf; C, couch grass; M, mixed litter; CG, control group); Arabic numerals represent different incubation periods (1, pre-incubation; 2, mid-incubation; 3, post-incubation).
