# Supplementary material for: Incubation determines favorable microbial communities in Chinese alligator nests
Source: Front Microbiol. 2022 Oct 13;13:983808. doi: 10.3389/fmicb.2022.983808 (PMC9606745; doi:10.3389/fmicb.2022.983808)
Supplement: Supplementary file 4 [file Table_4.DOCX]

**Table S4.** Summary of alpha diversity estimators for bacterial and fungal communities of three different nest material composition during different incubation periods

| Bacteria | Group  name | Richness estimates (Mean ± SE) | | | Diversity estimates (Mean ± SE) | | Good  coverage | PD whole  tree |
| --- | --- | --- | --- | --- | --- | --- | --- | --- |
|  |  | Observed species | Chao1 | ACE | Shannon | Simpson |  |  |
|  | B1 | 3472±455 | 3972±491 | 4140±500 | 9.318±0.731 | 0.988±0.013 | 0.982 | 209.343 |
|  | B2 | 4109±230 | 4700±222 | 4869±275 | 9.978±0.321 | 0.996±0.003 | 0.978 | 259.081 |
|  | B3 | 3936±181 | 4474±172 | 4707±225 | 9.913±0.160 | 0.996±0.000 | 0.979 | 240.328 |
|  | C1 | 3474±198 | 3969±265 | 4103±255 | 9.576±0.200 | 0.996±0.001 | 0.982 | 208.609 |
|  | C2 | 4035±205 | 4579±267 | 4716±283 | 10.037±0.191 | 0.997±0.000 | 0.98 | 254.516 |
|  | C3 | 4095±182 | 4693±211 | 4829±228 | 10.008±0.240 | 0.996±0.002 | 0.979 | 252.045 |
|  | M1 | 3233±228 | 3720±261 | 3859±283 | 9.166±0.479 | 0.991±0.009 | 0.983 | 196.218 |
|  | M2 | 3959±273 | 4524±296 | 4667±303 | 9.855±0.420 | 0.995±0.004 | 0.98 | 246.128 |
|  | M3 | 4139±205 | 4725±225 | 4898±237 | 9.995±0.382 | 0.995±0.006 | 0.979 | 257.311 |
|  | CG1 | 3694±165 | 4246±192 | 4427±205 | 9.293±0.461 | 0.987±0.008 | 0.98 | 227.808 |
|  | CG2 | 3685±198 | 4213±195 | 4379±273 | 9.591±0.300 | 0.995±0.002 | 0.98 | 227.656 |
|  | CG3 | 3634±166 | 4163±189 | 4326±196 | 9.451±0.174 | 0.993±0.003 | 0.981 | 218.13 |
| Fungi | B1 | 534±73 | 671±98 | 704±93 | 3.781±0.884 | 0.766±0.179 | 0.995 | 181.395 |
|  | B2 | 568±204 | 755±293 | 786±285 | 4.177±1.172 | 0.821±0.118 | 0.995 | 197.397 |
|  | B3 | 446±99 | 571±117 | 595±121 | 3.492±1.064 | 0.724±0.191 | 0.996 | 170.66 |
|  | C1 | 681±155 | 830±171 | 860±174 | 5.407±0.702 | 0.924±0.036 | 0.995 | 215.367 |
|  | C2 | 572±91 | 751±134 | 773±138 | 4.163±1.249 | 0.785±0.199 | 0.995 | 201.294 |
|  | C3 | 469±161 | 602±206 | 624±204 | 3.612±1.256 | 0.746±0.237 | 0.996 | 175.489 |
|  | M1 | 606±177 | 758±182 | 780±186 | 4.708±1.102 | 0.879±0.085 | 0.995 | 197.736 |
|  | M2 | 636±208 | 806±248 | 849±260 | 4.077±1.088 | 0.793±0.144 | 0.994 | 233.798 |
|  | M3 | 352±142 | 456±179 | 477±186 | 2.649±1.456 | 0.566±0.282 | 0.997 | 139.983 |
|  | CG1 | 357±118 | 460±149 | 488±166 | 3.631±0.772 | 0.804±0.082 | 0.997 | 122.156 |
|  | CG2 | 603±32 | 762±38 | 798±45 | 4.500±0.267 | 0.872±0.005 | 0.995 | 207.603 |
|  | CG3 | 369±49 | 472±83 | 487±70 | 3.701±0.660 | 0.830±0.082 | 0.997 | 138.788 |

The letters in group ID represents nest material composition (B, bamboo leaf; C, couch grass; M, mixed litter; CG, control group); Arabic numerals represent different incubation periods (1, pre-incubation; 2, mid-incubation; 3, post-incubation).
