# Supplementary material for: Incubation determines favorable microbial communities in Chinese alligator nests
Source: Front Microbiol. 2022 Oct 13;13:983808. doi: 10.3389/fmicb.2022.983808 (PMC9606745; doi:10.3389/fmicb.2022.983808)
Supplement: Supplementary file 5 [file Table_5.DOCX]

**Table S5.** Comparison of alpha diversity estimators between different incubation periods and nest material composition based on the Wilcoxon rank-sum test

| Pairwised  comparison | | ACE | | Chao1 | | observed_species | | shannon | | Simpson | |
| --- | --- | --- | --- | --- | --- | --- | --- | --- | --- | --- | --- |
|  |  | Difference | pvalue | Difference | pvalue | Difference | pvalue | Difference | pvalue | Difference | pvalue |
| Bacteria | B1 - B2 | -67.625 | **0.000** | -72.000 | **0.000** | -69.750 | **0.000** | -54.813 | **0.002** | -29.125 | 0.149 |
|  | B1 - B3 | -50.875 | **0.002** | -44.875 | **0.004** | -46.000 | **0.003** | -40.938 | **0.019** | -26.500 | 0.189 |
|  | B2 - B3 | 16.750 | 0.289 | 27.125 | 0.079 | 23.750 | 0.116 | 13.875 | 0.421 | 2.625 | 0.896 |
|  | C1 - C2 | -62.778 | **0.000** | -65.444 | **0.000** | -69.667 | **0.000** | -59.222 | **0.000** | -21.833 | 0.250 |
|  | C1 - C3 | -76.444 | **0.000** | -80.556 | **0.000** | -76.889 | **0.000** | -53.278 | **0.001** | -19.722 | 0.299 |
|  | C2 - C3 | -13.667 | 0.359 | -15.111 | 0.298 | -7.222 | 0.611 | 5.944 | 0.715 | 2.111 | 0.911 |
|  | M1 - M2 | -72.385 | **0.000** | -76.923 | **0.000** | -76.346 | **0.000** | -64.308 | **0.000** | -38.462 | **0.016** |
|  | M1 - M3 | -99.923 | **0.000** | -99.769 | **0.000** | -99.808 | **0.000** | -82.923 | **0.000** | -53.154 | **0.001** |
|  | M2 - M3 | -27.538 | 0.027 | -22.846 | 0.060 | -23.462 | 0.048 | -18.615 | 0.170 | -14.692 | 0.352 |
|  | CG1 - CG2 | 1.750 | 0.937 | 3.750 | 0.863 | 1.875 | 0.930 | -19.875 | 0.415 | -36.875 | 0.196 |
|  | CG1 - CG3 | 9.500 | 0.670 | 11.000 | 0.613 | 7.125 | 0.738 | -2.875 | 0.906 | -10.625 | 0.709 |
|  | CG2 - CG3 | 7.750 | 0.728 | 7.250 | 0.739 | 5.250 | 0.805 | 17.000 | 0.486 | 26.250 | 0.357 |
|  | B1 - C1 | 12.208 | 0.426 | 9.319 | 0.533 | 9.639 | 0.510 | -1.340 | 0.936 | -10.986 | 0.574 |
|  | B1 - M1 | 26.644 | 0.062 | 25.721 | 0.064 | 26.519 | 0.051 | 22.630 | 0.146 | 21.317 | 0.239 |
|  | C1 - M1 | 14.436 | 0.292 | 16.402 | 0.220 | 16.880 | 0.197 | 23.970 | 0.110 | 32.303 | 0.065 |
|  | B2 - C2 | 17.056 | 0.267 | 15.875 | 0.289 | 9.722 | 0.507 | -5.750 | 0.731 | -3.694 | 0.850 |
|  | B2 - M2 | 21.885 | 0.124 | 20.798 | 0.134 | 19.923 | 0.142 | 13.135 | 0.397 | 11.981 | 0.508 |
|  | C2 - M2 | 4.829 | 0.724 | 4.923 | 0.712 | 10.201 | 0.435 | 18.885 | 0.208 | 15.675 | 0.369 |
|  | B3 - C3 | -13.361 | 0.384 | -26.361 | 0.079 | -21.250 | 0.148 | -13.681 | 0.415 | -4.208 | 0.829 |
|  | B3 - M3 | -22.404 | 0.115 | -29.173 | **0.036** | -27.288 | **0.045** | -19.356 | 0.213 | -5.337 | 0.768 |
|  | C3 - M3 | -9.043 | 0.509 | -2.812 | 0.833 | -6.038 | 0.644 | -5.675 | 0.704 | -1.128 | 0.948 |
| Fungi | B1 - B2 | -9.750 | 0.591 | -9.875 | 0.589 | -6.000 | 0.742 | -14.500 | 0.477 | -10.188 | 0.625 |
|  | B1 - B3 | 24.321 | 0.197 | 22.536 | 0.234 | 25.821 | 0.172 | 8.696 | 0.680 | 15.768 | 0.465 |
|  | B2 - B3 | 34.071 | 0.071 | 32.411 | 0.088 | 31.821 | 0.093 | 23.196 | 0.273 | 25.955 | 0.229 |
|  | C1 - C2 | 9.778 | 0.568 | 9.778 | 0.570 | 19.500 | 0.257 | 45.333 | **0.020** | 41.944 | **0.034** |
|  | C1 - C3 | 46.333 | **0.008** | 47.444 | **0.007** | 52.944 | **0.002** | 63.389 | **0.001** | 55.889 | **0.005** |
|  | C2 - C3 | 36.556 | **0.034** | 37.667 | **0.030** | 33.444 | 0.053 | 18.056 | 0.348 | 13.944 | 0.478 |
|  | M1 - M2 | -10.769 | 0.450 | -8.000 | 0.577 | -5.692 | 0.690 | 22.692 | 0.157 | 29.615 | 0.071 |
|  | M1 - M3 | 54.923 | **0.000** | 56.385 | **0.000** | 58.808 | **0.000** | 59.385 | **0.000** | 61.423 | **0.000** |
|  | M2 - M3 | 65.692 | **0.000** | 64.385 | **0.000** | 64.500 | **0.000** | 36.692 | **0.023** | 31.808 | 0.053 |
|  | CG1 - CG2 | -65.750 | **0.011** | -66.750 | **0.011** | -67.250 | **0.010** | -36.750 | 0.204 | -20.125 | 0.495 |
|  | CG1 - CG3 | 7.250 | 0.778 | 2.750 | 0.915 | 5.375 | 0.835 | -4.500 | 0.876 | -9.375 | 0.750 |
|  | CG2 - CG3 | 73.000 | **0.005** | 69.500 | **0.008** | 72.625 | **0.005** | 32.250 | 0.264 | 10.750 | 0.715 |
|  | B1 - C1 | -28.472 | 0.108 | -30.861 | 0.084 | -33.917 | 0.057 | -59.875 | **0.003** | -52.931 | **0.010** |
|  | B1 - M1 | -12.712 | 0.436 | -15.673 | 0.340 | -14.212 | 0.385 | -30.875 | 0.094 | -32.452 | 0.084 |
|  | C1 - M1 | 15.761 | 0.318 | 15.188 | 0.338 | 19.705 | 0.213 | 29.000 | 0.103 | 20.479 | 0.258 |
|  | B2 - C2 | -8.944 | 0.612 | -11.208 | 0.528 | -8.417 | 0.634 | -0.042 | 0.998 | -0.799 | 0.969 |
|  | B2 - M2 | -13.731 | 0.401 | -13.798 | 0.401 | -13.904 | 0.396 | 6.317 | 0.730 | 7.351 | 0.694 |
|  | C2 - M2 | -4.786 | 0.761 | -2.590 | 0.870 | -5.487 | 0.728 | 6.359 | 0.719 | 8.150 | 0.652 |
|  | B3 - C3 | -6.460 | 0.724 | -5.952 | 0.746 | -6.794 | 0.711 | -5.183 | 0.801 | -12.810 | 0.542 |
|  | B3 - M3 | 17.890 | 0.294 | 18.176 | 0.289 | 18.775 | 0.272 | 19.813 | 0.301 | 13.203 | 0.499 |
|  | C3 - M3 | 24.350 | 0.124 | 24.128 | 0.129 | 25.568 | 0.107 | 24.996 | 0.159 | 26.013 | 0.151 |

Numbers in bold denote a significant difference (p < 0.05). Grey: Pairwised comparison between different nest material composition but incubation period is identical. The letters in group ID represents nest material composition (B, bamboo leaf; C, couch grass; M, mixed litter; CG, control group); Arabic numerals represent different incubation periods (1, pre-incubation; 2, mid-incubation; 3, post-incubation).
