# Supplementary material for: Incubation determines favorable microbial communities in Chinese alligator nests
Source: Front Microbiol. 2022 Oct 13;13:983808. doi: 10.3389/fmicb.2022.983808 (PMC9606745; doi:10.3389/fmicb.2022.983808)
Supplement: Supplementary file 6 [file Table_6.DOCX]

**Table S6.** Comparison of microbial community similarity during incubation using ANOSIM and the Wilcoxon rank-sum test

| Pairwised  comparison | | unweighted unifrac | | weighted unifrac | | Anosim | |
| --- | --- | --- | --- | --- | --- | --- | --- |
|  |  | Difference | p-value | Difference | p-value | R-value | P-value |
| Bacteria | B1 - B2 | -86.679 | 0.072 | 0.786 | 0.987 | 0.885 | **0.002** |
|  | B1 - B3 | 99.571 | **0.039** | 221.857 | **0.000** | 0.975 | **0.001** |
|  | B2 - B3 | 186.250 | **0.000** | 221.071 | **0.000** | 0.433 | **0.005** |
|  | C1 - C2 | -106.750 | **0.012** | -286.694 | **0.000** | 0.986 | **0.001** |
|  | C1 - C3 | 29.306 | 0.490 | -50.917 | 0.244 | 1.000 | **0.001** |
|  | C2 - C3 | 136.056 | **0.001** | 235.778 | **0.000** | 0.300 | **0.002** |
|  | M1 - M2 | -166.026 | **0.000** | -103.654 | **0.001** | 0.832 | **0.001** |
|  | M1 - M3 | -70.359 | **0.015** | 26.026 | 0.381 | 0.999 | **0.001** |
|  | M2 - M3 | 95.667 | **0.001** | 129.679 | **0.000** | 0.352 | **0.001** |
|  | CG1 - CG2 | -112.667 | 0.278 | 151.500 | 0.157 | 0.563 | **0.019** |
|  | CG1 - CG3 | 316.333 | **0.002** | 274.833 | **0.010** | 0.833 | **0.027** |
|  | CG2 - CG3 | 429.000 | **0.000** | 123.333 | 0.249 | 0.958 | **0.024** |
|  | B1 - C1 | 63.472 | 0.162 | 323.044 | **0.000** | 0.118 | **0.061** |
|  | B1 - M1 | 45.205 | 0.254 | 106.866 | **0.009** | 0.161 | **0.045** |
|  | C1 - M1 | -18.267 | 0.614 | -216.177 | **0.000** | 0.087 | 0.124 |
|  | B2 - C2 | 43.401 | 0.339 | 35.563 | 0.447 | 0.216 | **0.043** |
|  | B2 - M2 | -34.142 | 0.389 | 2.427 | 0.953 | 0.343 | **0.005** |
|  | C2 - M2 | -77.543 | **0.033** | -33.137 | 0.375 | 0.295 | **0.004** |
|  | B3 - C3 | -6.794 | 0.881 | 50.270 | 0.282 | 0.053 | **0.259** |
|  | B3 - M3 | -124.725 | **0.002** | -88.965 | **0.030** | 0.048 | **0.262** |
|  | C3 - M3 | -117.932 | **0.001** | -139.235 | **0.000** | -0.056 | 0.778 |
| Fungi | B1 - B2 | -98.536 | **0.021** | 19.036 | 0.713 | 0.055 | 0.231 |
|  | B1 - B3 | -5.560 | 0.904 | -169.810 | **0.003** | 0.199 | **0.012** |
|  | B2 - B3 | 92.976 | **0.043** | -188.845 | **0.001** | 0.001 | 0.441 |
|  | C1 - C2 | -59.056 | 0.115 | -60.750 | 0.183 | 0.488 | **0.001** |
|  | C1 - C3 | -205.472 | **0.000** | -5.917 | 0.897 | 0.654 | **0.001** |
|  | C2 - C3 | -146.417 | **0.000** | 54.833 | 0.230 | -0.020 | 0.559 |
|  | M1 - M2 | -111.167 | **0.000** | -124.641 | **0.000** | 0.420 | **0.001** |
|  | M1 - M3 | -265.218 | **0.000** | -181.833 | **0.000** | 0.406 | **0.001** |
|  | M2 - M3 | -154.051 | **0.000** | -57.192 | 0.065 | 0.146 | **0.004** |
|  | CG1 - CG2 | 313.000 | **0.001** | 180.500 | 0.107 | 0.115 | 0.242 |
|  | CG1 - CG3 | 50.500 | 0.582 | 196.333 | 0.079 | -0.052 | 0.586 |
|  | CG2 - CG3 | -262.500 | **0.004** | 15.833 | 0.887 | -0.135 | 0.723 |
|  | B1 - C1 | 77.329 | 0.054 | 93.567 | 0.055 | 0.750 | **0.001** |
|  | B1 - M1 | -7.124 | 0.839 | 92.364 | **0.031** | 0.734 | **0.001** |
|  | C1 - M1 | -84.453 | **0.009** | -1.203 | 0.975 | 0.536 | **0.002** |
|  | B2 - C2 | 116.810 | **0.004** | 13.782 | 0.778 | 0.465 | **0.001** |
|  | B2 - M2 | -19.755 | 0.573 | -51.312 | 0.229 | 0.356 | **0.002** |
|  | C2 - M2 | -136.564 | **0.000** | -65.094 | 0.096 | 0.151 | **0.029** |
|  | B3 - C3 | -122.583 | **0.005** | 257.460 | **0.000** | 0.381 | **0.001** |
|  | B3 - M3 | -266.782 | **0.000** | 80.341 | 0.092 | 0.221 | **0.024** |
|  | C3 - M3 | -144.199 | **0.000** | -177.120 | **0.000** | 0.115 | 0.075 |

Numbers in bold denote a significant difference (p < 0.05). Grey: Pairwised comparison between different nest material composition but incubation period is identical. The letters in group ID represents nest material composition (B, bamboo leaf; C, couch grass; M, mixed litter; CG, control group); Arabic numerals represent different incubation periods (1, pre-incubation; 2, mid-incubation; 3, post-incubation).
