# Supplementary figures and images for: Incubation determines favorable microbial communities in Chinese alligator nests
Source: Front Microbiol. 2022 Oct 13;13:983808. doi: 10.3389/fmicb.2022.983808 (PMC9606745; doi:10.3389/fmicb.2022.983808)

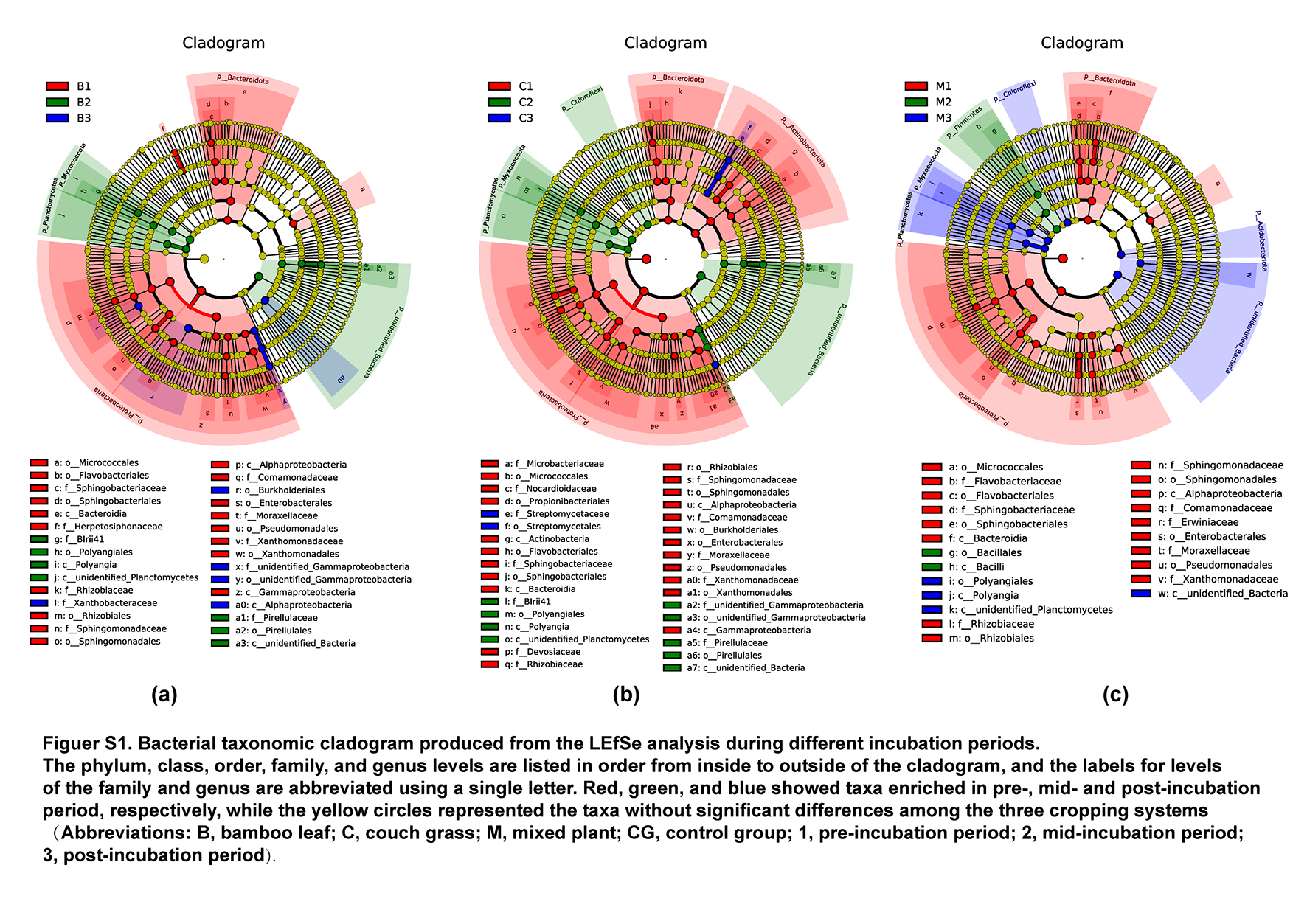

Supplement: Supplementary file 7 [file Image_1.TIF]

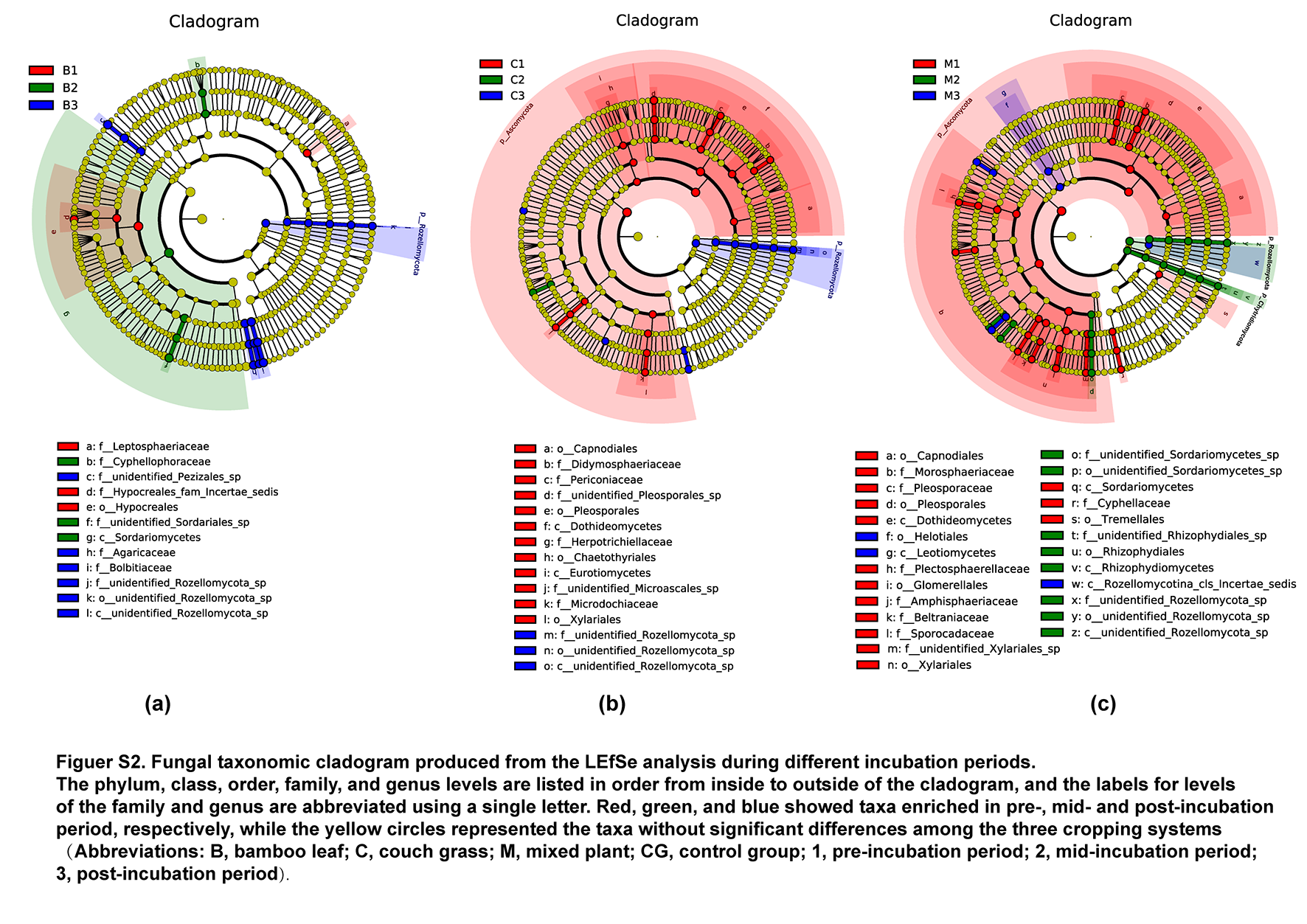

Supplement: Supplementary file 8 [file Image_2.TIF]

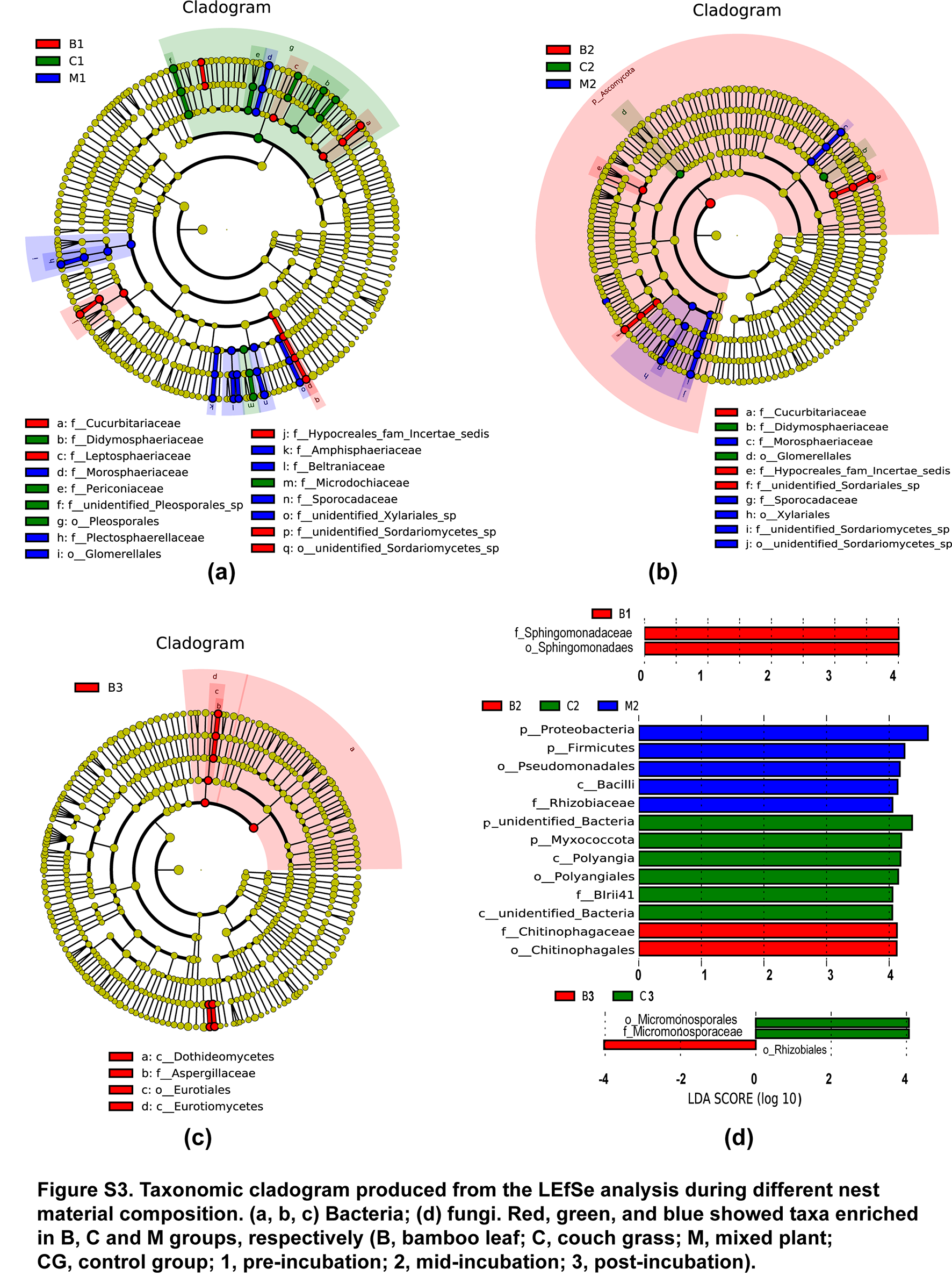

Supplement: Supplementary file 9 [file Image_3.TIF]
